# Establishing a core outcome set for mucopolysaccharidoses (MPS) in children: study protocol for a rapid literature review, candidate outcomes survey, and Delphi surveys

**DOI:** 10.1186/s13063-021-05791-8

**Published:** 2021-11-17

**Authors:** Alison H. Howie, Kylie Tingley, Michal Inbar-Feigenberg, John J. Mitchell, Nancy J. Butcher, Martin Offringa, Maureen Smith, Kim Angel, Jenifer Gentle, Alexandra Wyatt, Philippe M. Campeau, Alicia Chan, Pranesh Chakraborty, Farah El Turk, Eva Mamak, Aizeddin Mhanni, Becky Skidmore, Rebecca Sparkes, Sylvia Stockler, Beth K. Potter, in collaboration with the INFORM RARE Network

**Affiliations:** 1 School of Epidemiology and Public Health, University of Ottawa, Room 101, 600 Peter Morand Crescent, Ottawa ON, Canada, Ottawa, ON K1G 5Z3 Canada; 2 The Hospital for Sick Children, Toronto, ON Canada; 3 McGill University Health Centre, Montreal, QC Canada; 4 Child Health Evaluative Sciences, The Hospital for Sick Children Research Institute, Toronto, ON Canada; 5 Department of Psychiatry, University of Toronto, Toronto, ON Canada; 6 Department of Pediatrics, University of Toronto, Toronto, ON Canada; 7 Patient Partner, Canadian Organization for Rare Disorders, Ottawa, ON Canada; 8 Canadian MPS Society, Vancouver, BC Canada; 9 Patient/Family Partner, Vancouver, BC Canada; 10 Department of Pediatrics, CHU Sainte-Justine and Université de Montréal, Montreal, QC Canada; 11 Department of Medical Genetics, University of Alberta, Edmonton, AB Canada; 12 Children’s Hospital of Eastern Ontario, Ottawa, ON Canada; 13 Department of Pediatrics, University of Ottawa, Ottawa, ON Canada; 14 Department of Psychology, The Hospital for Sick Children, Toronto, ON Canada; 15 Department of Pediatrics and Child Health, and Department of Biochemistry and Medical Genetics, Max Rady College of Medicine, Rady Faculty of Health Sciences, University of Manitoba, Winnipeg, MB Canada; 16 Ottawa Hospital Research Institute, Ottawa, ON Canada; 17 Department of Medical Genetics and Pediatrics, University of Calgary, Calgary, AB Canada; 18 Biochemical Diseases, BC Children’s Hospital, Vancouver, BC Canada

**Keywords:** Outcomes research, Mucopolysaccharidoses, Pediatrics, Rare diseases

## Abstract

**Background:**

Mucopolysaccharidoses (MPS) are a group of inherited metabolic diseases characterized by chronic, progressive multi-system manifestations with varying degrees of severity. Disease-modifying therapies exist to treat some types of MPS; however, they are not curative, underscoring the need to identify and evaluate co-interventions that optimize functioning, participation in preferred activities, and quality of life. A Canadian pediatric MPS registry is under development and may serve as a platform to launch randomized controlled trials to evaluate such interventions. To promote the standardized collection of patient/family-reported and clinical outcomes considered important to patients/families, health care providers (HCPs), and policymakers, the choice of outcomes to include in the registry will be informed by a core outcome set (COS). We aim to establish a patient-oriented COS for pediatric MPS using a multi-stakeholder approach.

**Methods:**

In step 1 of the six-step process to develop the COS, we will identify relevant outcomes through a rapid literature review and candidate outcomes survey. A two-phase screening approach will be implemented to identify eligible publications, followed by extraction of outcomes and other pre-specified data elements. Simultaneously, we will conduct a candidate outcomes survey with children with MPS and their families to identify outcomes most important to them. In step 2, HCPs experienced in treating patients with MPS will be invited to review the list of outcomes generated in step 1 and identify additional clinically relevant outcomes. We will then ask patients/families, HCPs, and policymakers to rate the outcomes in a set of Delphi Surveys (step 3), and to participate in a subsequent consensus meeting to finalize the COS (step 4). Step 5 involves establishing a set of outcome measurement instruments for the COS. Finally, we will disseminate the COS to knowledge users (step 6).

**Discussion:**

The proposed COS will inform the choice of outcomes to include in the MPS registry and, more broadly, promote the standardized collection of patient-oriented outcomes for pediatric MPS research. By involving patients/families from the earliest stage of the research, we will ensure that the COS will be relevant to those who will ultimately benefit from the research.

**Trial registration:**

PROSPERO CRD42021267531, COMET

**Supplementary Information:**

The online version contains supplementary material available at 10.1186/s13063-021-05791-8.

## Background

### Introduction

Mucopolysaccharidoses (MPS) are a group of inherited metabolic disorders (IMDs) that have an autosomal recessive inheritance pattern, except for MPS II which is X-linked [1]. Overall birth prevalence estimates for MPS vary by country/region and range from 1.04 to 4.8 per 100,000 live births [2]. Mucopolysaccharidoses are a heterogenous group of conditions. The clinical manifestations of MPS often begin early in life and are chronic, progressive, and typically involve multiple organ systems. There are also milder subtypes that may be diagnosed later in life, for example, some cases of attenuated MPS I. Common clinical symptoms and signs include dysostosis multiplex, muscular, connective tissue and airway problems, vision loss, hearing loss, cardiovascular manifestations, and organomegaly, among others [1, 3]. Many subtypes involve the central nervous system, leading to neurocognitive impairment, neurodevelopmental regression, epilepsy, and neurologic dysfunctions [3, 4]. A better understanding of the pathophysiology and natural history of MPS, substantial progress in the development and availability of MPS-specific treatments (e.g., enzyme replacement therapy (ERT) and hematopoietic stem cell transplant (HSCT) for some subtypes), and advances in supportive symptom-based treatments have led to improved patient prognosis over the last several years [5, 6]. While none of the available treatments offer a cure for the disease, disease-modifying treatments (drugs, surgical, and gene therapies) continue to emerge [6, 7]. Given that these emerging therapies are not curative, there is a need to identify additional co-interventions and approaches to care that optimize functioning, participation in preferred activities [8], and quality of life based on the priorities of patients and their families.

Despite being the gold standard for primary clinical research that aims to evaluate interventions, explanatory randomized controlled trials (RCTs) have been criticized for generating results that may be of little importance to patients in the real world, given their reliance on controlled settings and highly selected patients [9–11]. Pragmatic RCTs aim to overcome this barrier by measuring the effectiveness of interventions in “real-world” settings; that is, in diverse groups of patients, followed in routine care, measuring stakeholder-relevant outcomes [12]. One means of conducting pragmatic RCTs is through a registry, defined as a collection of data obtained from numerous sources, including electronic health records, patient-reported data, and administrative data for clinical, research, administrative, and/or policy purposes [13]. Registries can be established to measure health services use among a broad population or to follow patients defined by a particular disease or condition [13]. Registry-based RCTs have gained prominence due to their potential ability to generate results with real-world applicability [13]. Among the numerous proposed advantages to registry-based trials are efficiencies in recruitment, the potential for more complete and efficient long-term follow-up via the registry, and a lower need for active trial-specific data collection from patients (to the extent that trial outcomes and other data are obtained from the registry) [13–16].

While pragmatic trials may offer real-world applicability, issues persist in outcome measurement and reporting. Trial outcomes and outcome measurement instruments are often highly variable across studies, thus precluding comparison and synthesis of results, and are often selectively reported, whereby positive or “significant” outcomes are more likely to be emphasized in published manuscripts [17]. The standardized selection and measurement of outcomes, established via consensus processes with relevant stakeholders and used across all studies in a particular disease area, aim to address these concerns. The Core Outcome Measures in Effectiveness Trials (COMET) Initiative was developed to promote the standardized collection of outcome measurements through core outcomes sets (COSs), defined as an agreed-upon standard set of outcome measures for a particular disease or condition [18]. The COMET Initiative encourages the collection of COSs to reduce research waste and to facilitate decision-making processes by all health care users [18]. By measuring the same outcomes, irrespective of the intervention (whether it be a drug, a therapy session, a diet, or an exercise regimen), direct comparisons are possible across a wide range of studies. This allows for a larger number of studies to be directly compared and synthesized in systematic reviews, which are often used to guide policy decision making and clinical practice guidelines.

COSs have been developed for a variety of pediatric conditions, including phenylketonuria (PKU), medium-chain acyl-CoA dehydrogenase deficiency (MCAD) deficiency, gastroschisis, and otitis media [19–21]. A COS is currently in development for head, neck, and respiratory disease in MPS II [22]. However, no COSs have been developed, or are in development, for MPS more broadly. While each MPS subtype presents unique manifestations and these change with age, several patient-oriented outcomes such as pain, difficulty sleeping, and reduced quality of life are experienced by many children with MPS across subtypes and age groups.

INFORM RARE is a Canadian pediatric rare disease network that is developing a patient-oriented longitudinal registry for children with MPS to serve as a platform for registry-based randomized trials and observational studies. To inform data collection for our MPS registry and to improve outcome selection and reporting in studies of pediatric MPS more broadly, we aim to establish a COS containing outcomes important for all children with MPS. As the daily impact of MPS on children and their families is substantial, including for those receiving disease-modifying therapies, our COS will emphasize outcomes deemed important to patients and families and relevant to child and family functioning, participation in preferred activities, and quality of life. The COS will also include a set of additional outcomes that may be relevant in specific age groups or for children diagnosed with specific MPS subtypes or groups of MPS subtypes (e.g., those with neuronopathic involvement) [4] (Table 1).

**Table 1:** Summary of MPS subtypes

MPS subtype	Common name(s)	Enzyme deficiency^**c**^	Neuronopathic involvement^**d**^
I			
IH^a,b^	Hurler syndrome	α-L-iduronidase	+
IA^a^	Attenuated MPSI, Hurler-Scheie syndrome, Scheie syndrome	α-L-iduronidase	+/-
II			
IIA^a^	Hunter (severe) syndrome	Iduronate sulfatase	+
IIB^a^	Hunter (mild) syndrome	Iduronate sulfatase	+/-
III			
IIIA	Sanfilippo syndrome A	Heparan N-sulfatase	+
IIIB	Sanfilippo syndrome B	α-N-acetyl-glucosaminidase	+
IIIC	Sanfilippo syndrome C	Acetyl CoA:α glucosaminide acetlytransferase	+
IIID	Sanfilippo syndrome D	N-acetylglucosamine 6-sulfatase	+
IV			
IVA^a^	Morquio syndrome A	N-acetylgalactosamine 6-sulfatase (GALNS)	-
IVB	Morquio syndrome B	β-galactosidase	+/-
VI^a^	Maroteaux-Lamy syndrome	N-acetylgalactosamine 4-sulfatase (Arylsulfatase B)	-
VII^a,b^	Sly syndrome	β-glucuronidase	+
IX	Natowicz syndrome	Hyaluronidase	-

*MPS* mucopolysaccharidosis

^a^Enzyme replacement therapy (ERT) is frequently used for this subtype of MPS

^b^Hematopoietic stem cell transplantation (HSCT) is used for this subtype of MPS

^c^Information taken from Zhou and colleagues 2020 [23]

^d^+ Typical for this subtype, −atypical for this subtype, and +/− occurs in some cases or depends on how neuronopathic involvement is defined

The objectives of this study are as follows:

i) Identify all outcomes measured in recent intervention studies and recommended for measurement in recent guidelines for pediatric MPS;
ii) Identify additional outcomes important to pediatric patients with MPS and their family members and to health care providers with expertise in MPS care;
iii) Achieve consensus across stakeholders (patients and their family members, health care providers, and methodologists) on a COS to be measured in future studies of MPS;
iv) Identify and recommend outcome measurement instruments used for each outcome in the COS.

## Methods

This protocol is reported according to the Core Outcome Set-STAndardised Protocol (COS-STAP) Statement [24].

**Fig. 1:**
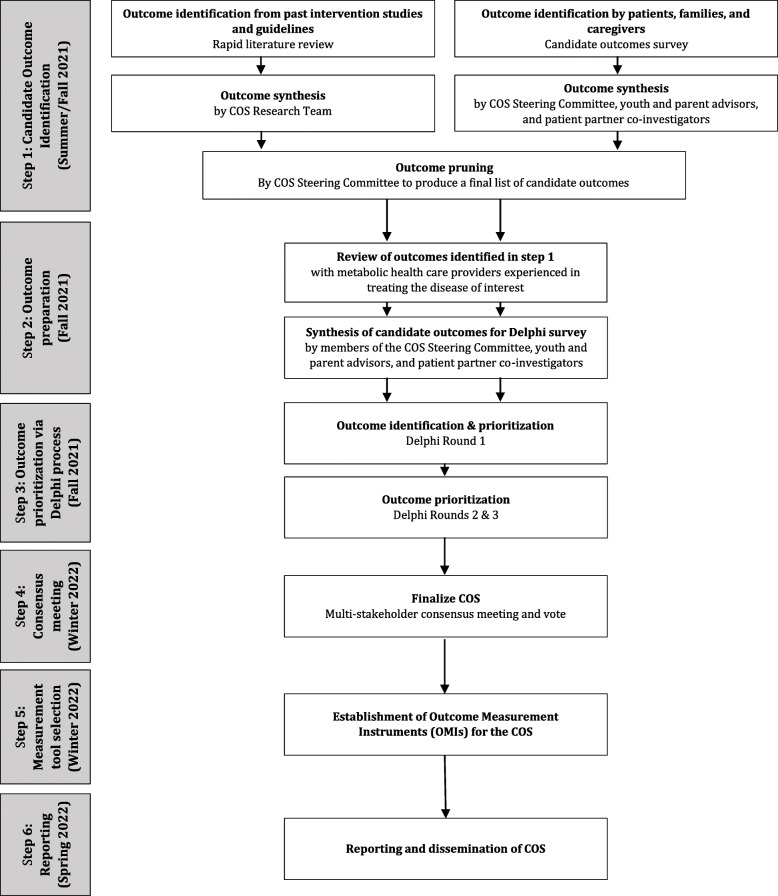
Outline of Core Outcome Set (COS) development process (adapted from Monga et al., 2020) [26]

**Fig. 2:**
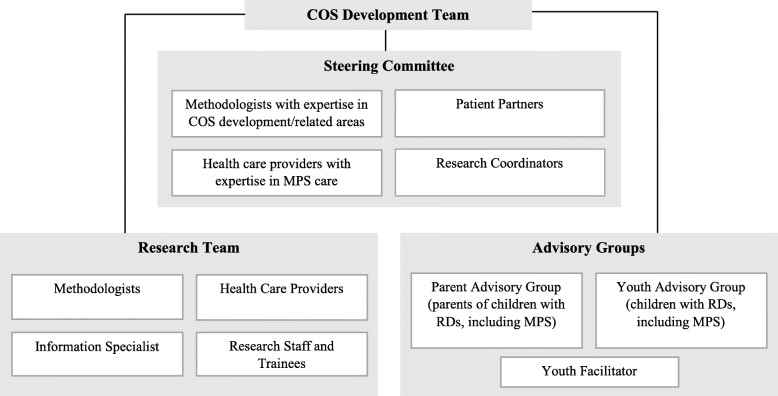
Core outcome set development team (adapted from Monga et al., 2020) [26]

Figure 1 outlines our proposed six-step process for developing the COS. This process was established based on previous COS development literature (including our own COSs for PKU and MCAD deficiency) [25, 26] and following the COMET guidelines [27]. Figure 2 outlines the various groups involved in the development of the COS.

### Patient engagement

There is evidence that outcomes deemed important by researchers and health care providers differ from those deemed important by patients and families [27]. As such, COMET recommends that patients and families are included in the development of a COS [27]. Patients and families are key members of the INFORM RARE research team as principal investigators and co-investigators and have contributed to the conceptualization of this protocol.  This core group of patient partners will be involved in all steps of the COS development process. Engagement with a Youth Advisory Group and Parent Advisory Group, which include three youth with MPS and three parents of children with MPS, respectively, will ensure that individuals with lived experience of MPS have an opportunity to provide feedback at key points in the COS development process. Further, we will engage with Canadian and international patient organizations to allow them the opportunity to contribute to the development of the COS, and to raise awareness about the COS to facilitate dissemination once complete.

### Step 1a: Rapid literature review

A rapid literature review [28] will be conducted to identify (i) what outcomes were measured in recent or ongoing clinical trials and/or recommended in recent guidelines for pediatric MPS and (ii) what instruments are being used to measure such outcomes. The review will be streamlined in terms of the time frame and study design (described below) to obtain the most relevant results in a resource-efficient manner [28].

The rapid review protocol was developed following the Preferred Reporting Items for Systematic Reviews and Meta-Analyses statement for protocols (PRISMA-P) (Additional file 1) and  registered with PROSPERO (CRD42021267531; submitted July 12, 2021). All post hoc amendments relative to the methods described below will be documented and acknowledged in the final report.

#### Literature search to identify relevant studies

An experienced information specialist (B Skidmore) developed the search strategy following an iterative process in consultation with the review team. A second senior information specialist peer-reviewed the MEDLINE strategy prior to execution using the PRESS checklist [29]. Any suggested edits from the PRESS were reviewed and incorporated where appropriate.

Using the multifile option as well as the deduping tool in Ovid, we searched Ovid MEDLINE® ALL, including Epub Ahead of Print, In-Process & Other Non-Indexed Citations, Embase Classic + Embase, and the Cochrane Central Register of Controlled Trials. We also searched CINAHL using the Ebsco platform. We downloaded and deduped results using EndNote 9.3.3 (Clarivate) and uploaded to Covidence (Veritas Health Innovation). Searches contained a mixture of controlled vocabulary (e.g., “Mucopolysaccharidosis”) and free-text terms (e.g., “Hunter syndrome,” “ARSB deficiency,” “alpha-L-iduronidase deficiency”). Study design filters for randomized and non-randomized controlled trials and clinical practice guidelines were applied where appropriate. We removed animal-only records and limited results to the publication years 2011 to the present. All searches were performed on May 16, 2021. Search results were downloaded and deduped using EndNote 9.3.3 (Clarivate) and uploaded to Covidence (Veritas Health Innovation). The full strategies appear in Additional file 1.

We performed a gray literature search of guideline registries listed in CADTH’s Grey Matters, including Turning Research Into Practice (TRIP), and also searched ClinicalTrials.gov.

#### Study eligibility criteria

The purpose of the COS is to inform the choice of outcomes to be included in a pediatric MPS registry, which will then be used to launch registry-based trials and observational studies. Thus, we aim to identify a broad list of candidate outcomes from recent studies. The study eligibility criteria will be defined by PICOTS: population, intervention/exposure, comparator, outcomes, time frame, and study design (Table 2) [30]. The objective of the review is to identify all outcomes related to pediatric MPS; thus, no restrictions will be placed on outcomes. Further, to capture a broad range of outcomes, no restrictions will be applied to interventions/exposures and to comparator groups. In keeping with the “rapid” review, we will restrict our search based on the time frame and study design. We have selected the last 10 years as our time frame to identify the most current outcomes. As new therapies are being developed, so too are new ways to measure the success of these therapies; thus, looking at the most recent publications will yield the most relevant outcomes. Our search will further be limited to non-animal intervention studies (published and/or protocols) and guidelines related to MPS published in English to identify the most salient outcomes in MPS research and to reduce the number of articles for screening. For the purposes of this review, a guideline is defined as (i) a clinical practice guideline from a professional association or from a governmental organization at a regional or higher level of geography that provides formal recommendations for clinical practice (i.e., guidelines published by individual research teams, hospitals, or similarly small jurisdictions will be excluded), (ii) an article from a multi-disciplinary group of MPS experts making broad recommendations for the clinical management of MPS, or (iii) an article from a multi-disciplinary group of MPS experts making recommendations about endpoints to measure in research studies of MPS. To be included, guidelines must specify the specific outcomes recommended for monitoring patients and/or for evaluating interventions at an individual patient or aggregate level. Given the exceptional rarity of certain subtypes of MPS, we may conduct additional post hoc searches (by widening the time frame and/or including non-interventional studies and potentially liberalizing our definition of a guideline) for certain very rare subtypes should our initial search yield few results.

**Table 2:** Study eligibility criteria defined by PICOTS

PICOTS component	Description
Population	Children (18 years or younger) diagnosed with MPS (all subtypes)
Interventions/exposures	No restrictions on interventions and exposures
Comparators	No restrictions on comparators
Outcomes	No restrictions on outcomes
Time frame	2011-2021
Study design	(i) Non-animal intervention studies of MPS; and (ii) Guidelines for the clinical management of MPS and other guidelines and recommendations related to MPS *outcomes*

*MPS* mucopolysaccharidosis, *PICOTS* population, interventions/exposures, comparators, outcomes, time frame, and study design

#### Process of study screening and selection

Articles identified through the bibliographic and gray literature searches will be synthesized, removing duplicates, and uploaded into Covidence. We will follow a two-phase approach to screening. In phase one, titles and abstracts will be screened by two independent reviewers. Articles considered eligible by both reviewers will move to phase two, while articles considered ineligible by both reviewers will be removed; conflicts will be resolved during consensus discussions involving a third team member. The same strategy will be applied in phase two for full-text screening. The list of the guidelines that were deemed eligible or potentially eligible in phase one, along with the decision of whether to include them in phase two, will be shared with a small multi-disciplinary COS Steering Committee (Fig. 2) who will make final judgments about excluded guidelines. Further, clinicians on the Steering Committee will be asked to identify any additional guidelines that they feel are important to inform care. Such studies will automatically move to full-text extraction. The screening process will be summarized in a PRISMA flow diagram, with explanations for exclusions in phase two.

The two-phase screening process will be piloted to ensure that the process is reliable. Twenty articles and ten articles will be piloted for phases one and two, respectively. If agreement between reviewers is poor, modifications to the screening form, available in Additional file 1, may be made and a second pilot screen will be undertaken until the agreement is adequate.

#### Data collection

Data will be extracted from included articles by one reviewer and verified by another in a data extraction form in Microsoft Excel (Additional file 2). The extraction will focus on the PICOTS, with a detailed focus on outcomes and outcome measurement instruments as well as characteristics of the population studied (e.g., child ages, MPS subtypes). In addition, basic study characteristics, including title, journal, and country of publication, will be extracted. Similar to the screening process, the data extraction form will be piloted on a sample of 10 articles. Reviewers will discuss the utility of the extraction form and make any necessary changes before proceeding with the remaining articles.

#### Analysis and presentation of summarized findings

A list of unique outcomes will be generated by scanning the outcomes identified in the literature and removing duplicates. Results will be analyzed descriptively. Outcomes will be summarized by publication year, age range of study participants, and MPS subtypes.

### Step 1 b: Candidate outcomes survey

In parallel with the rapid review, we will conduct a short survey to identify outcomes that are most important to children with MPS and their families.

#### Survey participants and recruitment strategy

Eligible participants will include children and young adults aged 14–25 years diagnosed with MPS (any subtype) and parents/caregivers to children (18 years and younger) with MPS residing in Canada. We will work with relevant patient advocacy organizations (e.g., Canadian Society for Mucopolysaccharide and Related Diseases Inc. (also operates under the name “Canadian MPS Society”), The Isaac Foundation) to distribute study invitations. Given that we would like to recruit a broad sample of individuals, we will use a snowball sampling technique whereby study invitation recipients will be encouraged to circulate the invitation to others in their contact network(s) who may be interested in participating. In addition, the survey information link will be posted on social media platforms. All survey responses submitted before the study deadline will be analyzed.

#### Study development, data collection, and analysis

A short web-based survey was developed in collaboration with a patient-partner lead investigator (M Smith) and reviewed by patient partner co-investigators (K Angel, J Gentle) as well as both Youth and Parent Advisory groups; this led to key changes to ensure that relevant concepts were incorporated clearly and that accessible language was used. Participants will be invited to describe up to three outcomes that they feel are important and may warrant inclusion in a COS for pediatric MPS. We will use REDCap (Research Electronic Data Capture) hosted at the Children’s Hospital of Eastern Ontario Research Institute to administer the survey. A copy of the candidate outcomes survey is included as Additional file 4.

The results from the survey will be reviewed by the research team and analyzed descriptively. Outcomes reported by respondents will be synthesized by the COS Steering Committee, the Youth and Parent Advisory Groups, and patient partner co-investigators to produce a list of *patient and family generated outcomes* that will be merged with the *rapid review generated outcomes* to produce a final list of candidate outcomes. The Steering Committee, in consultation with the full study team, will generate a list of unique outcomes by scanning the candidate outcomes list and combining those that are identified by different names but that measure the same or a very similar concept (outcome pruning) to facilitate subsequent steps. For example, academic achievement in English, academic achievement in math, and academic achievement in science may be combined into one outcome entitled “academic achievement/school performance.” Outcome pruning will also include the exclusion of outcomes from the rapid review that are not patient-centered and that are deemed irrelevant by the COS Steering Committee. Further, the outcomes will be summarized by MPS subtype and by age within these groupings.

### Step 2: Outcome preparation

We will survey a purposive sample of specialist health care providers to elicit their opinions about whether the list of candidate outcomes contains all potentially important outcomes from a clinical care perspective and, if not, to identify and incorporate additional outcomes. We will also ask for their views on the organization of the candidate outcomes with respect to grouping of MPS subtypes and age categories.

#### Survey participants and recruitment strategy

Eligible participants will include metabolic physicians as well as other specialist and subspecialist physicians and allied health professionals who have experience treating patients diagnosed with an MPS disorder. We will work with members of our research network to generate a list of potentially eligible health care providers on an international scale. We will circulate study invitations using the publicly available contact information. Snowball sampling will be used. All survey responses submitted before the study deadline will be analyzed.

#### Survey development, data collection, and analysis

A brief web-based survey will be developed to gauge whether the candidate outcomes list (developed in step 1) is comprehensive. Participants will be presented with the list of outcomes and will be asked if they feel that all potentially relevant outcomes (from a clinical care perspective) have been identified. Participants who select “no” will have the opportunity to list additional outcomes that will be incorporated into the final list of outcomes that will be presented in the Delphi survey. Again, we will use REDCap to administer the survey and to store the survey results. The results from the survey will be reviewed by the study team and analyzed descriptively.

The outcomes identified from steps 1 and 2 will be synthesized by the COS Steering Committee, youth and parent advisors, and patient partner co-investigators. The final list of outcomes will be grouped into categories based on their “core area” defined by the Outcome Measures in Rheumatology (OMERACT) 2.0 Filter adapted for pediatric COSs: death, life impact, resource use, pathophysiological manifestations, and growth and development [31, 32]. This outcome list will form the basis for outcome prioritization via a Delphi survey (step 3). Outcomes that were identified by patients and parents/caregivers will be flagged in the Delphi survey.

### Step 3: Delphi consensus surveys

We will conduct a Delphi survey to begin to establish consensus on outcomes for inclusion in a final COS for pediatric MPS. Our approach follows methods recommended for COS development [27], and the consensus approach used by our team to develop COSs for MCAD deficiency and PKU [19, 25].

#### Delphi participants and recruitment strategy

Eligible participants will include individuals and parents/caregivers of individuals diagnosed with MPS, health care providers, and health policy decision makers. All participants will be required to reside in Canada.

Participants will receive study invitations by email from patient advocacy organizations, professional associations, contacts in their professional network, or directly from INFORM RARE using publicly available information. Given that we would like to recruit a broad sample of individuals, snowball sampling will be used. In addition, we will post the survey information and survey link on social media. Based on our previous experience developing COSs for rare pediatric diseases, we anticipate between 50 and 75 participants for the Delphi survey.

#### Delphi questionnaire development, data collection, and analysis

In collaboration with patient partner co-investigators, youth and parent advisors, and the COS Steering Committee, we will plan two rounds of Delphi surveys, with the potential for a third round if warranted [27]. Based on established recommendations [27], the Delphi questionnaires will be written in lay language for all stakeholder groups (professionals as well as patients and their family members). We will use three strategies to maximize the number of participants completing the surveys and to reduce attrition. First, as participants are more likely to take part in surveys if they are well-structured and not overly lengthy, we will divide the surveys into sections defined by the core areas to allow participants to consider one set of outcomes at a time [27] and, as mentioned above, we will complete outcome pruning to combine similar outcomes. Second, we will carefully select the dates that the surveys will be administered, avoiding holidays and summer months when individuals may be less inclined to participate [27]. Lastly, we will minimize the amount of time between each round to avoid having participants lose interest [27]. Each round will be open for 3 weeks, followed by a 3-week period to analyze the results and develop the next round [27].

In round one, participants will be presented with the final list of outcomes from step 2, along with definitions for each outcome, and asked to rate the importance of each outcome on a 9-point Likert scale, as recommended by the Grading of Recommendations Assessment, Development and Evaluation (GRADE) Working Group [27, 33]. Outcomes that are judged to be of limited importance will be awarded a score between 1 and 3, outcomes that are important but not critical 4–6, and outcomes that are critical for inclusion in the COS 7–9 [27]. Participants will have the opportunity, at the end of the survey, to list any additional outcomes that they feel are important to consider for inclusion in the COS. Survey results will be stored in REDCap and analyzed descriptively (mean, median, percentage of scores in each category) by the stakeholder group.

All outcomes from round one will be retained in the second round, irrespective of how they were scored [27]. Further, all additional outcomes identified by participants will be included in round two. Only individuals who participated in round one (including partial responders) will be invited to round two. Participants will be presented with their own scores for each outcome, and, to encourage consensus both within and between groups, scores summarized separately by stakeholder group [27]. Participants will be asked to re-consider how they scored each outcome, taking into account feedback from other participants. However, participants will be reminded that there is no pressure to change their scores from round one. Round two results will be analyzed descriptively by stakeholder group. To determine consensus, the following rules will be applied: outcomes that are deemed “critical” by at least 70% of participants and “of limited importance” by fewer than 15% will move on to the next stage [27]. Conversely, outcomes that are deemed “of limited importance” by at least 70% of participants and “critical” by fewer than 15% will be excluded [27]. While we do not expect that consensus will be reached on all outcomes, if too few outcomes are agreed on to be “critical” or “of limited importance,” the study team may decide to conduct a third round of Delphi surveys.

### Step 4: Final consensus meeting

Following the final round of the Delphi survey, a virtual consensus meeting will be held to arrive at a final set of outcomes that will comprise the COS for pediatric MPS. All Delphi participants will have the opportunity to attend the meeting and vote on the outcomes. Patients and families will receive training prior to the consensus meeting to ensure that they can participate meaningfully in the process. We will use a nominal group technique to allow all participant’s perspectives to be considered. We anticipate that the COS will contain a maximum of nine outcomes per group (number of groups to be determined but we anticipate having a list of broad MPS outcomes, as well as groups defined by age and by MPS subtype and/or subtype category, e.g., based on neuronopathic involvement) [19, 20].

### Step 5: Establishment of outcome measurement instruments for the COS

Following the consensus meeting, the COS Steering Committee will establish a set of outcome measurement instruments for the COS. A list of outcome measurement instruments identified in the rapid review will be compiled and reviewed by the COS Steering Committee, who will have the opportunity to suggest additional instruments. We will determine the relevance, feasibility, cost, validity, and reliability of each instrument following guidance from COSMIN et al. [34]. The decision to recommend each instrument will be based on balancing trade-offs between the aforementioned characteristics (e.g., an instrument that has high reliability but is seldom used in practice is unlikely to be recommended). The patient partner co-investigators and the Youth and Parent Advisory Groups will contribute to discussions about outcome measurement instruments, particularly for outcomes best measured by patient/parent/caregiver reports. The final COS and potential outcome measurement instruments will be circulated to international colleagues and patient organizations for feedback before they are finalized.

### Step 6: Dissemination

Knowledge users for the COS may include, but are not limited to, research funders, journal editors, patients and families, policymakers, health care providers, and trialists [35]. Some of these users are included on our research team as part of our integrated knowledge translation strategy (patients and families, health care providers, and methodologists). To further reach this group and to engage additional knowledge users, we plan to publish the results of the rapid review, as well as the final COS in open-access academic journals. Moreover, we intend to present our results at webinars and conferences. An infographic will be created to summarize our findings in lay terminology that will be shared with patient organizations (e.g., The Canadian MPS Society), clinician networks (e.g., The Garrod Association), and policy networks (e.g., through CADTH) for further distribution [36].

## Discussion

Rigorous studies are required to evaluate interventions for children with MPS, including new disease-modifying therapies and co-interventions that aim to optimize functioning, participation in preferred activities, and quality of life. Inconsistencies in outcome selection, measurement, and reporting lead to difficulties in interpreting the evidence base from clinical trials in general [17]. These difficulties are heightened for rare diseases such as MPS, for which high-quality evidence is in short supply [37, 38].

We describe a multi-stakeholder approach to developing a COS for pediatric MPS. The perspectives of patients and families with lived experiences of MPS are integrated throughout the process. The goal of the COS is to promote the standardized measurement of relevant and meaningful outcomes in a Canadian pediatric MPS registry, which will serve as a platform to rapidly launch a variety of studies to answer patient-oriented questions. These studies include registry-based RCTs which offer efficiencies with respect to recruitment, data collection, and long-term follow-up [13–16]. In addition to benefitting participants in the Canadian registry, the COS will also contribute to greater harmonization and transparency in outcome selection and reporting in trials internationally, as well as a focus on outcomes that are most meaningful to patients and families. Randomized controlled trials in the field of MPS could report on the core outcomes as a minimum, in addition to any trial-specific outcomes that are prioritized.

### Status

At the time of submission of this manuscript, the search strategy for the rapid review has been peer-reviewed and implemented; and screening against inclusion/exclusion criteria is underway.

## Supplementary information

**Additional file 1.** PRISMA, search strategy, screening forms

**Additional file 2.** Draft data extraction form

**Additional file 3.** COS-STAP checklist

**Additional file 4.** Candidate outcomes survey

## Data Availability

Not applicable (no datasets have been generated as part of this protocol).

## Abbreviations

CIMDRN: Canadian Inherited Metabolic Diseases Network
CNDR: Canadian Neuromuscular Disease Registry
COS: Core outcome set
COS-STAP: Core Outcome Set-STAndardised Protocol
COMET: Core Outcome Measures in Effectiveness Trials
ERT: Enzyme replacement therapy
GRADE: Grading of Recommendations Assessment, Development and Evaluation
HCP: Health care provider
HSCT: Hematopoietic stem cell transplant
IMD: Inherited metabolic disease
MCAD: Medium-chain Acyl-CoA dehydrogenase
MPS: Mucopolysaccharidosis
PKU: Phenylketonuria
PRESS: Peer Review of Electronic Search Strategies
PRISMA: Preferred Reporting Items for Systematic Reviews and Meta-Analyses
RCT: Randomized controlled trial
REDCap: Research Electronic Data Capture
TRIP: Turning research into practice
