# Correction to: Establishing a core outcome set for mucopolysaccharidoses (MPS) in children: study protocol for a rapid literature review, candidate outcomes survey, and Delphi surveys

**DOI:** 10.1186/s13063-021-05893-3

**Published:** 2021-12-09

**Authors:** Alison H. Howie, Kylie Tingley, Michal Inbar-Feigenberg, John J. Mitchell, Nancy J. Butcher, Martin Offringa, Maureen Smith, Kim Angel, Jenifer Gentle, Alexandra Wyatt, Philippe M. Campeau, Alicia Chan, Pranesh Chakraborty, Farah El Turk, Eva Mamak, Aizeddin Mhanni, Becky Skidmore, Rebecca Sparkes, Sylvia Stockler, Beth K. Potter, in collaboration with the INFORM RARE Network

**Affiliations:** 1 School of Epidemiology and Public Health, University of Ottawa, Room 101, 600 Peter Morand Crescent, Ottawa, ON K1G 5Z3 Canada; 2 The Hospital for Sick Children, Toronto, ON Canada; 3 McGill University Health Centre, Montreal, QC Canada; 4 Child Health Evaluative Sciences, The Hospital for Sick Children Research Institute, Toronto, ON Canada; 5 Department of Psychiatry, University of Toronto, Toronto, ON Canada; 6 Department of Pediatrics, University of Toronto, Toronto, ON Canada; 7 Patient Partner, Canadian Organization for Rare Disorders, Ottawa, ON Canada; 8 Canadian MPS Society, Vancouver, BC Canada; 9 Patient/Family Partner, Vancouver, BC Canada; 10 Department of Pediatrics, CHU Sainte-Justine and Université de Montréal, Montreal, QC Canada; 11 Department of Medical Genetics, University of Alberta, Edmonton, AB Canada; 12 Children’s Hospital of Eastern Ontario, Ottawa, ON Canada; 13 Department of Pediatrics, University of Ottawa, Ottawa, ON Canada; 14 Department of Psychology, The Hospital for Sick Children, Toronto, ON Canada; 15 Department of Pediatrics and Child Health, and Department of Biochemistry and Medical Genetics, Max Rady College of Medicine, Rady Faculty of Health Sciences, University of Manitoba, Winnipeg, MB Canada; 16 Ottawa Hospital Research Institute, Ottawa, ON Canada; 17 Department of Medical Genetics and Pediatrics, University of Calgary, Calgary, AB Canada; 18 Biochemical Diseases, BC Children’s Hospital, Vancouver, BC Canada

**Correction to: Trials 22, 816 (2021)**

**https://doi.org/10.1186/s13063-021-05791-8**

Following the publication of the original article [1], we were notified of the below affiliation corrections:

- Nancy J. Butcher is affiliated to 4 & 5 instead of 6
- Martin Offringa is affiliated to 2, 4 & 6 instead of only 6
- Farah El Turk is affiliated to 3 & 10, instead of just 3

The original article has been corrected.
