# Supplementary material for: Establishing a core outcome set for mucopolysaccharidoses (MPS) in children: study protocol for a rapid literature review, candidate outcomes survey, and Delphi surveys
Source: Trials. 2021 Nov 17;22:816. doi: 10.1186/s13063-021-05791-8 (PMC8600749; doi:10.1186/s13063-021-05791-8)
Supplement: Supplementary file 1 — Additional file 1. PRISMA, search strategy, screening forms [file 13063_2021_5791_MOESM1_ESM.docx]

**1.1 PRISMA-P Checklist**

**PRISMA-P (Preferred Reporting Items for Systematic review and Meta-Analysis Protocols) 2015 checklist: recommended items to address in a systematic review protocol***

| Section and topic | Item No | Checklist item | Page # in protocol |
| --- | --- | --- | --- |
| ADMINISTRATIVE INFORMATION | | |  |
| Title: |  |  |  |
| Identification | 1a | Identify the report as a protocol of a systematic review | 1 |
| Update | 1b | If the protocol is for an update of a previous systematic review, identify as such | n/a |
| Registration | 2 | If registered, provide the name of the registry (such as PROSPERO) and registration number | 3 |
| Authors: |  |  |  |
| Contact | 3a | Provide name, institutional affiliation, e-mail address of all protocol authors; provide physical mailing address of corresponding author | 1-2 |
| Contributions | 3b | Describe contributions of protocol authors and identify the guarantor of the review | 22 |
| Amendments | 4 | If the protocol represents an amendment of a previously completed or published protocol, identify as such and list changes; otherwise, state plan for documenting important protocol amendments | 9 |
| Support: |  |  |  |
| Sources | 5a | Indicate sources of financial or other support for the review | 22 |
| Sponsor | 5b | Provide name for the review funder and/or sponsor | 22 |
| Role of sponsor or funder | 5c | Describe roles of funder(s), sponsor(s), and/or institution(s), if any, in developing the protocol | 22 |
| INTRODUCTION | | |  |
| Rationale | 6 | Describe the rationale for the review in the context of what is already known | 4-7 |
| Objectives | 7 | Provide an explicit statement of the question(s) the review will address with reference to participants, interventions, comparators, and outcomes (PICO) | 7 |
| METHODS | | |  |
| Eligibility criteria | 8 | Specify the study characteristics (such as PICO, study design, setting, time frame) and report characteristics (such as years considered, language, publication status) to be used as criteria for eligibility for the review | 10-11, Table 2 |
| Information sources | 9 | Describe all intended information sources (such as electronic databases, contact with study authors, trial registers or other grey literature sources) with planned dates of coverage | 9-10 |
| Search strategy | 10 | Present draft of search strategy to be used for at least one electronic database, including planned limits, such that it could be repeated | Appendix 1 |
| Study records: |  |  | 11 |
| Data management | 11a | Describe the mechanism(s) that will be used to manage records and data throughout the review |  |
| Selection process | 11b | State the process that will be used for selecting studies (such as two independent reviewers) through each phase of the review (that is, screening, eligibility and inclusion in meta-analysis) | 11-12 |
| Data collection process | 11c | Describe planned method of extracting data from reports (such as piloting forms, done independently, in duplicate), any processes for obtaining and confirming data from investigators | 12 |
| Data items | 12 | List and define all variables for which data will be sought (such as PICO items, funding sources), any pre-planned data assumptions and simplifications | 12, Additional file 2 |
| Outcomes and prioritization | 13 | List and define all outcomes for which data will be sought, including prioritization of main and additional outcomes, with rationale | 12 |
| Risk of bias in individual studies | 14 | Describe anticipated methods for assessing risk of bias of individual studies, including whether this will be done at the outcome or study level, or both; state how this information will be used in data synthesis | n/a |
| Data synthesis | 15a | Describe criteria under which study data will be quantitatively synthesised | n/a |
|  | 15b | If data are appropriate for quantitative synthesis, describe planned summary measures, methods of handling data and methods of combining data from studies, including any planned exploration of consistency (such as I^2^, Kendall’s τ) | n/a |
|  | 15c | Describe any proposed additional analyses (such as sensitivity or subgroup analyses, meta-regression) | n/a |
|  | 15d | If quantitative synthesis is not appropriate, describe the type of summary planned | 12 |
| Meta-bias(es) | 16 | Specify any planned assessment of meta-bias(es) (such as publication bias across studies, selective reporting within studies) | n/a |
| Confidence in cumulative evidence | 17 | Describe how the strength of the body of evidence will be assessed (such as GRADE) | n/a |

*** It is strongly recommended that this checklist be read in conjunction with the PRISMA-P Explanation and Elaboration (cite when available) for important clarification on the items. Amendments to a review protocol should be tracked and dated. The copyright for PRISMA-P (including checklist) is held by the PRISMA-P Group and is distributed under a Creative Commons Attribution Licence 4.0.**

*From: Shamseer L, Moher D, Clarke M, Ghersi D, Liberati A, Petticrew M, Shekelle P, Stewart L, PRISMA-P Group. Preferred reporting items for systematic review and meta-analysis protocols (PRISMA-P) 2015: elaboration and explanation. BMJ. 2015 Jan 2;349(jan02 1):g7647.*

**1.2 Final Search Strategy (following peer review)**

INFORM – RARE – MPS

Final Strategy

2021 May 16

Ovid Multifile

Database: Embase Classic+Embase <1947 to 2021 May 14> , Ovid MEDLINE(R) ALL <1946 to May 14, 2021>, EBM Reviews - Cochrane Central Register of Controlled Trials <April 2021>

Search Strategy:

--------------------------------------------------------------------------------

1 exp Mucopolysaccharidosis/ (20992)

2 (mucopolysaccharidos#s or gargolylis* or gargoylis*).tw,kf. (13495)

3 ((alpha-l-iduronidase or iduronidase) adj2 (disease? or syndrome? or deficien*)).tw,kf. (294)

4 ((Hurler* or Hurler-Scheie* or Pfaundler-Hurler* or Scheie* or Schleie*) adj2 (disease? or syndrome?)).tw,kf. (2935)

5 (helmholtz harrington adj2 (disease? or syndrome?)).tw,kf. (0)

6 (chondroosteodysplas* or chondro-osteodysplas* or chondroosteodystroph* or chondro-osteodystroph* or chondroosteoplas* or chondro-osteoplas* or dysostos#s multiplex).tw,kf. (804)

7 (lipochondrodystroph* or lipo-chondrodystroph*).tw,kf. (435)

8 mckusick 25280.tw,kf. (16)

9 ((I2S or iduronate* or "iduronate 2" or sulfoiduronate* or sulfo-iduronate*) adj2 deficien*).tw,kf. (273)

10 (Hunter* adj2 (disease? or syndrome?)).tw,kf. (2546)

11 hunter* glossit#s.tw,kf. (23)

12 ((heparan sulfate or heparan sulphate or heparan sulfamidase or heparan sulphamidase or heparitin sulfate or heparitin sulphate or heparan-alpha-glucosaminide n-acetyltransferase) adj2 (deficien* or storage disease?)).tw,kf. (263)

13 heparitinuri*.tw,kf. (0)

14 mckusick 30990.tw,kf. (0)

15 ((sanfilippo* or san filippo*) adj2 deficien*).tw,kf. (27)

16 ((n-acetyl-alpha-d-glucosaminidase or n-acetylglucosamine-6-sulfat* or n-acetylglucosamine-6-sulphat* or NAGLU) adj2 deficien*).tw,kf. (105)

17 ((polydystrophic* or poly-dystrophic*) adj2 (dwarf* or oligophreni* or oligo-phreni*)).tw,kf. (37)

18 ((acetyl-CoA alpha-glucosaminide n-acetyltransferase or heparan-alpha-glucosaminide n-acetyltransferase or HGSNAT) adj2 deficien*).tw,kf. (25)

19 ((n-acetylglucosamine-6-sulfate sulfatase or n-acetylglucosamine-6-sulphate sulphatase or glucosamine n-acetyl-6-sulfatase or glucosamine n-acetyl-6-sulphatase or GNS) adj2 deficien*).tw,kf. (22)

20 (morquio* adj2 (disease? or syndrome?)).tw,kf. (1978)

21 ((galactosamine-6-sulfatase or galactosamine-6-sulphatase or n-acetylgalactosamine-6-sulfate sulfatase or n-acetylgalactosamine-6-sulphate sulphatase or GALNS) adj2 deficien*).tw,kf. (177)

22 (kerato sulfatur* or kerato sulphatur* or keratosulfatur* or keratosulphatur*).tw,kf. (10)

23 ((osteochondrodystroph* or osteo-chondrodystroph*) adj1 deformans).tw,kf. (15)

24 familial osseous dystroph*.tw,kf. (2)

25 (eccentro?steochondrodysplas* or eccentro-osteochondrodysplas* or eccentro-osteo-chondrodysplasia* or eccentroosteo-chondrodysplas*).tw,kf. (136)

26 (maroteaux lamy adj2 (disease? or syndrome?)).tw,kf. (723)

27 ((arylsulfatase b or arylsulphatase b or n-acetylgalactosamine-4-sulfat* or n-acetylgalactosamine-4-sulphat* or ARSB) adj2 deficien*).tw,kf. (288)

28 mckusick 25320.tw,kf. (0)

29 (sly adj2 (disease? or syndrome?)).tw,kf. (228)

30 ((beta-glucuronidase or GUSB) adj2 deficien*).tw,kf. (457)

31 (scleromyxedem* or sclero-myxedem*).tw,kf. (925)

32 (lichen adj1 (fibromucinoidos* or fibro-mucinoidos* or myxedematos* or myxoedematos* or myxooedematos* or myxo-oedematos*)).tw,kf. (452)

33 ((mucinosis or mucinous*) adj3 (papular* or papulos*)).tw,kf. (453)

34 ((myxedema* or myxoedema* or myxooedema* or myxo-oedema*) adj3 (papular or papulos*)).tw,kf. (95)

35 (MPS 1? or MPS1? or MPS I? or MPSI? or "MPS1-H" or "MPSIH/S" or "MPS1H/S" or "MPS1-HS" or "MPS1-S" or MPSIS or MPS 2 or MPS2 or MPS II or MPSII or MPS 3? or MPS3? or MPS III? or MPSIII? or MPS 4? or MPS4? or MPS IV? or MPSIV? or MPS 6 or MPS6 or MPS VI or MPSVI or MPS 7 or MPS7 or MPS VII or MPSVII or MPS 9 or MPS9 or MPS IX or MPSIX).tw,kf. (15766)

36 or/1-35 [MPS] (34448)

37 exp Adult/ not (Adolescent/ or exp Child/ or exp Infant/) (14173618)

38 36 not 37 [ADULT-ONLY REMOVED] (29246)

39 exp Animals/ not Humans/ (16884742)

40 38 not 39 [ANIMAL-ONLY REMOVED] (22104)

41 limit 40 to yr="2011-current" (8810)

42 (controlled clinical trial or randomized controlled trial or pragmatic clinical trial or equivalence trial).pt. (1225827)

43 "Clinical Trials as Topic"/ (311671)

44 exp "Controlled Clinical Trials as Topic"/ (371070)

45 (randomi#ed or randomi#ation? or randomly or RCT or placebo*).tw,kf. (3651523)

46 ((singl* or doubl* or trebl* or tripl*) adj (mask* or blind* or dumm*)).tw,kf. (725913)

47 trial.ti. (924003)

48 or/42-47 [RCT FILTER] (4600148)

49 41 and 48 [RCTs] (494)

50 controlled clinical trial.pt. (186233)

51 Controlled Clinical Trial/ or Controlled Clinical Trials as Topic/ (573312)

52 (control* adj2 trial).tw,kf. (674038)

53 Non-Randomized Controlled Trials as Topic/ (12538)

54 (nonrandom* or non-random* or quasi-random* or quasi-experiment*).tw,kf. (154090)

55 (nRCT or non-RCT).tw,kf. (987)

56 Controlled Before-After Studies/ (226950)

57 (control* adj3 ("before and after" or "before after")).tw,kf. (804059)

58 Interrupted Time Series Analysis/ (219841)

59 time series.tw,kf. (75339)

60 (pre- adj5 post-).tw,kf. (329864)

61 (pretest adj5 posttest).tw,kf. (17114)

62 Historically Controlled Study/ (237400)

63 (control* adj2 study).tw,kf. (547559)

64 Control Groups/ (124519)

65 (control* adj2 group?).tw,kf. (1555496)

66 trial.ti. (924003)

67 or/50-66 [nRCT FILTER] (4348515)

68 41 and 67 [nRCTs] (417)

69 exp Clinical Pathways/ (16157)

70 exp Clinical Protocols/ (298896)

71 Consensus/ (93400)

72 exp Consensus Development Conference/ (36824)

73 exp Consensus Development Conferences as Topic/ (27457)

74 exp Guideline/ (35712)

75 Guidelines as Topic/ (457489)

76 Practice Guidelines as Topic/ (474951)

77 Health Planning Guidelines/ (105477)

78 (Guideline or Practice Guideline or Consensus Development Conference or Consensus Development Conference, NIH).pt. (45370)

79 (position statement* or policy statement* or practice parameter* or best practice*).tw,kf. (92705)

80 (standards or guideline or guidelines).ti,kf. (258958)

81 ((practice or treatment* or clinical) adj guideline*).ab. (116036)

82 (CPG or CPGs).ti. (13249)

83 consensus*.ti,kf. (61894)

84 consensus*.ab. /freq=2 (65764)

85 ((critical or clinical or practice) adj2 (path or paths or pathway or pathways or protocol*)).tw,kf. (59083)

86 recommendat*.ti,kf. (100357)

87 (overview? adj2 guideline?).tw,kf. (308)

88 or/69-87 [CPG FILTER] (1538391)

89 41 and 88 [CPGs] (227)

90 49 or 68 or 89 [ALL STUDY DESIGNS] (878)

91 90 use medall [MEDLINE RECORDS] (332)

92 exp mucopolysaccharidosis/ (20992)

93 (mucopolysaccharidos#s or gargolylis* or gargoylis*).tw,kw. (13693)

94 ((alpha-l-iduronidase or iduronidase) adj2 (disease? or syndrome? or deficien*)).tw,kw. (297)

95 ((Hurler* or Hurler-Scheie* or Pfaundler-Hurler* or Scheie* or Schleie*) adj2 (disease? or syndrome?)).tw,kw. (2989)

96 (helmholtz harrington adj2 (disease? or syndrome?)).tw,kw. (0)

97 (chondroosteodysplas* or chondro-osteodysplas* or chondroosteodystroph* or chondro-osteodystroph* or chondroosteoplas* or chondro-osteoplas* or dysostos#s multiplex).tw,kw. (838)

98 (lipochondrodystroph* or lipo-chondrodystroph*).tw,kw. (490)

99 mckusick 25280.tw,kw. (16)

100 ((I2S or iduronate* or "iduronate 2" or sulfoiduronate* or sulfo-iduronate*) adj2 deficien*).tw,kw. (273)

101 (Hunter* adj2 (disease? or syndrome?)).tw,kw. (2592)

102 hunter* glossit#s.tw,kw. (24)

103 ((heparan sulfate or heparan sulphate or heparan sulfamidase or heparan sulphamidase or heparitin sulfate or heparitin sulphate or heparan-alpha-glucosaminide n-acetyltransferase) adj2 (deficien* or storage disease?)).tw,kw. (264)

104 heparitinuri*.tw,kw. (0)

105 mckusick 30990.tw,kw. (0)

106 ((sanfilippo* or san filippo*) adj2 deficien*).tw,kw. (27)

107 ((n-acetyl-alpha-d-glucosaminidase or n-acetylglucosamine-6-sulfat* or n-acetylglucosamine-6-sulphat* or NAGLU) adj2 deficien*).tw,kw. (105)

108 ((polydystrophic* or poly-dystrophic*) adj2 (dwarf* or oligophreni* or oligo-phreni*)).tw,kw. (37)

109 ((acetyl-CoA alpha-glucosaminide n-acetyltransferase or heparan-alpha-glucosaminide n-acetyltransferase or HGSNAT) adj2 deficien*).tw,kw. (25)

110 ((n-acetylglucosamine-6-sulfate sulfatase or n-acetylglucosamine-6-sulphate sulphatase or glucosamine n-acetyl-6-sulfatase or glucosamine n-acetyl-6-sulphatase or GNS) adj2 deficien*).tw,kw. (22)

111 (morquio* adj2 (disease? or syndrome?)).tw,kw. (1994)

112 ((galactosamine-6-sulfatase or galactosamine-6-sulphatase or n-acetylgalactosamine-6-sulfate sulfatase or n-acetylgalactosamine-6-sulphate sulphatase or GALNS) adj2 deficien*).tw,kw. (178)

113 (kerato sulfatur* or kerato sulphatur* or keratosulfatur* or keratosulphatur*).tw,kw. (10)

114 ((osteochondrodystroph* or osteo-chondrodystroph*) adj1 deformans).tw,kw. (15)

115 familial osseous dystroph*.tw,kw. (2)

116 (eccentro?steochondrodysplas* or eccentro-osteochondrodysplas* or eccentro-osteo-chondrodysplasia* or eccentroosteo-chondrodysplas*).tw,kw. (158)

117 (maroteaux lamy adj2 (disease? or syndrome?)).tw,kw. (748)

118 ((arylsulfatase b or arylsulphatase b or n-acetylgalactosamine-4-sulfat* or n-acetylgalactosamine-4-sulphat* or ARSB) adj2 deficien*).tw,kw. (290)

119 mckusick 25320.tw,kw. (0)

120 (sly adj2 (disease? or syndrome?)).tw,kw. (244)

121 ((beta-glucuronidase or GUSB) adj2 deficien*).tw,kw. (463)

122 (scleromyxedem* or sclero-myxedem*).tw,kw. (941)

123 (lichen adj1 (fibromucinoidos* or fibro-mucinoidos* or myxedematos* or myxoedematos* or myxooedematos* or myxo-oedematos*)).tw,kw. (472)

124 ((mucinosis or mucinous*) adj3 (papular* or papulos*)).tw,kw. (468)

125 ((myxedema* or myxoedema* or myxooedema* or myxo-oedema*) adj3 (papular or papulos*)).tw,kw. (96)

126 (MPS 1? or MPS1? or MPS I? or MPSI? or "MPS1-H" or "MPSIH/S" or "MPS1H/S" or "MPS1-HS" or "MPS1-S" or MPSIS or MPS 2 or MPS2 or MPS II or MPSII or MPS 3? or MPS3? or MPS III? or MPSIII? or MPS 4? or MPS4? or MPS IV? or MPSIV? or MPS 6 or MPS6 or MPS VI or MPSVI or MPS 7 or MPS7 or MPS VII or MPSVII or MPS 9 or MPS9 or MPS IX or MPSIX).tw,kw. (15836)

127 or/92-126 [MPS] (34537)

128 exp adult/ not exp juvenile/ (8052810)

129 127 not 128 [ADULT-ONLY REMOVED] (31013)

130 exp animal/ or exp animal experimentation/ or exp animal model/ or exp animal experiment/ or nonhuman/ or exp vertebrate/ (55743169)

131 exp human/ or exp human experimentation/ or exp human experiment/ (43449265)

132 130 not 131 (12295760)

133 129 not 132 [ANIMAL-ONLY REMOVED] (27567)

134 limit 133 to yr="2011-current" (12712)

135 exp randomized controlled trial/ or controlled clinical trial/ (1467044)

136 clinical trial/ (1551744)

137 exp "controlled clinical trial (topic)"/ (210467)

138 (randomi#ed or randomi#ation? or randomly or RCT or placebo*).tw,kw. (3714210)

139 ((singl* or doubl* or trebl* or tripl*) adj (mask* or blind* or dumm*)).tw,kw. (754692)

140 trial.ti. (924003)

141 or/135-140 [RCT FILTER] (5192747)

142 134 and 141 [RCTs] (861)

143 controlled clinical trial/ (557793)

144 "controlled clinical trial (topic)"/ (11575)

145 (control* adj2 trial).tw,kw. (1026477)

146 (nonrandom* or non-random* or quasi-random* or quasi-experiment*).tw,kw. (155103)

147 (nRCT or non-RCT).tw,kw. (989)

148 (control* adj3 ("before and after" or "before after")).tw,kw. (804063)

149 time series analysis/ (29066)

150 time series.tw,kw. (76343)

151 pretest posttest control group design/ (561)

152 (pre- adj5 post-).tw,kw. (329907)

153 (pretest adj5 posttest).tw,kw. (20804)

154 controlled study/ (8138197)

155 (control* adj2 study).tw,kw. (957555)

156 control group/ (124418)

157 (control* adj2 group?).tw,kw. (1556694)

158 trial.ti. (924003)

159 or/143-158 [nRCT FILTER] (11191228)

160 134 and 159 [nRCTs] (2037)

161 exp practice guideline/ (623500)

162 (position statement* or policy statement* or practice parameter* or best practice*).tw,kw. (93271)

163 (standards or guideline or guidelines).ti,kw. (287621)

164 ((practice or treatment* or clinical) adj guideline*).ab. (116036)

165 (CPG or CPGs).ti. (13249)

166 consensus/ (93400)

167 consensus*.ti,kw. (68924)

168 consensus*.ab. /freq=2 (65764)

169 ((critical or clinical or practice) adj2 (path or paths or pathway or pathways or protocol*)).tw,kw. (65909)

170 recommendat*.ti,kw. (103226)

171 (overview? adj2 guideline?).tw,kw. (312)

172 or/161-171 [CPG FILTER] (1212464)

173 134 and 172 [CPGs] (307)

174 142 or 160 or 173 [ALL STUDY DESIGNS] (2684)

175 174 use emczd [EMBASE RECORDS] (2059)

176 exp Mucopolysaccharidosis/ (20992)

177 (mucopolysaccharidos#s or gargolylis* or gargoylis*).ti,ab,kw. (13693)

178 ((alpha-l-iduronidase or iduronidase) adj2 (disease? or syndrome? or deficien*)).ti,ab,kw. (297)

179 ((Hurler* or Hurler-Scheie* or Pfaundler-Hurler* or Scheie* or Schleie*) adj2 (disease? or syndrome?)).ti,ab,kw. (2989)

180 (helmholtz harrington adj2 (disease? or syndrome?)).ti,ab,kw. (0)

181 (chondroosteodysplas* or chondro-osteodysplas* or chondroosteodystroph* or chondro-osteodystroph* or chondroosteoplas* or chondro-osteoplas* or dysostos#s multiplex).ti,ab,kw. (838)

182 (lipochondrodystroph* or lipo-chondrodystroph*).ti,ab,kw. (490)

183 mckusick 25280.ti,ab,kw. (16)

184 ((I2S or iduronate* or "iduronate 2" or sulfoiduronate* or sulfo-iduronate*) adj2 deficien*).ti,ab,kw. (273)

185 (Hunter* adj2 (disease? or syndrome?)).ti,ab,kw. (2592)

186 hunter* glossit#s.ti,ab,kw. (24)

187 ((heparan sulfate or heparan sulphate or heparan sulfamidase or heparan sulphamidase or heparitin sulfate or heparitin sulphate or heparan-alpha-glucosaminide n-acetyltransferase) adj2 (deficien* or storage disease?)).ti,ab,kw. (264)

188 heparitinuri*.ti,ab,kw. (0)

189 mckusick 30990.ti,ab,kw. (0)

190 ((sanfilippo* or san filippo*) adj2 deficien*).ti,ab,kw. (27)

191 ((n-acetyl-alpha-d-glucosaminidase or n-acetylglucosamine-6-sulfat* or n-acetylglucosamine-6-sulphat* or NAGLU) adj2 deficien*).ti,ab,kw. (105)

192 ((polydystrophic* or poly-dystrophic*) adj2 (dwarf* or oligophreni* or oligo-phreni*)).ti,ab,kw. (37)

193 ((acetyl-CoA alpha-glucosaminide n-acetyltransferase or heparan-alpha-glucosaminide n-acetyltransferase or HGSNAT) adj2 deficien*).ti,ab,kw. (25)

194 ((n-acetylglucosamine-6-sulfate sulfatase or n-acetylglucosamine-6-sulphate sulphatase or glucosamine n-acetyl-6-sulfatase or glucosamine n-acetyl-6-sulphatase or GNS) adj2 deficien*).ti,ab,kw. (22)

195 (morquio* adj2 (disease? or syndrome?)).ti,ab,kw. (1994)

196 ((galactosamine-6-sulfatase or galactosamine-6-sulphatase or n-acetylgalactosamine-6-sulfate sulfatase or n-acetylgalactosamine-6-sulphate sulphatase or GALNS) adj2 deficien*).ti,ab,kw. (178)

197 (kerato sulfatur* or kerato sulphatur* or keratosulfatur* or keratosulphatur*).ti,ab,kw. (10)

198 ((osteochondrodystroph* or osteo-chondrodystroph*) adj1 deformans).ti,ab,kw. (15)

199 familial osseous dystroph*.ti,ab,kw. (2)

200 (eccentro?steochondrodysplas* or eccentro-osteochondrodysplas* or eccentro-osteo-chondrodysplasia* or eccentroosteo-chondrodysplas*).ti,ab,kw. (158)

201 (maroteaux lamy adj2 (disease? or syndrome?)).ti,ab,kw. (748)

202 ((arylsulfatase b or arylsulphatase b or n-acetylgalactosamine-4-sulfat* or n-acetylgalactosamine-4-sulphat* or ARSB) adj2 deficien*).ti,ab,kw. (290)

203 mckusick 25320.ti,ab,kw. (0)

204 (sly adj2 (disease? or syndrome?)).ti,ab,kw. (244)

205 ((beta-glucuronidase or GUSB) adj2 deficien*).ti,ab,kw. (463)

206 (scleromyxedem* or sclero-myxedem*).ti,ab,kw. (941)

207 (lichen adj1 (fibromucinoidos* or fibro-mucinoidos* or myxedematos* or myxoedematos* or myxooedematos* or myxo-oedematos*)).ti,ab,kw. (472)

208 ((mucinosis or mucinous*) adj3 (papular* or papulos*)).ti,ab,kw. (468)

209 ((myxedema* or myxoedema* or myxooedema* or myxo-oedema*) adj3 (papular or papulos*)).ti,ab,kw. (96)

210 (MPS 1? or MPS1? or MPS I? or MPSI? or "MPS1-H" or "MPSIH/S" or "MPS1H/S" or "MPS1-HS" or "MPS1-S" or MPSIS or MPS 2 or MPS2 or MPS II or MPSII or MPS 3? or MPS3? or MPS III? or MPSIII? or MPS 4? or MPS4? or MPS IV? or MPSIV? or MPS 6 or MPS6 or MPS VI or MPSVI or MPS 7 or MPS7 or MPS VII or MPSVII or MPS 9 or MPS9 or MPS IX or MPSIX).ti,ab,kw. (15834)

211 or/176-210 [MPS] (34535)

212 limit 211 to yr="2011-current" (16716)

213 212 use cctr [CENTRAL RECORDS] (230)

214 91 or 175 or 213 [ALL DATABASES] (2621)

215 remove duplicates from 214 (2292) [TOTAL UNIQUE RECORDS]

216 215 use medall [MEDLINE UNIQUE RECORDS] (325)

217 215 use emczd [EMBASE UNIQUE RECORDS] (1841)

218 215 use cctr [CENTRAL UNIQUE RECORDS] (126)

***************************

CINAHL

| # | Query | Limiters/Expanders | Last Run Via | Results |
| --- | --- | --- | --- | --- |
| S78 | S46 OR S64 OR S77 | Expanders - Apply related words; Apply equivalent subjects  Search modes - Boolean/Phrase | Interface - EBSCOhost Research Databases  Search Screen - Advanced Search  Database - CINAHL | 62 |
| S77 | S40 AND S76 | Expanders - Apply related words; Apply equivalent subjects  Search modes - Boolean/Phrase | Interface - EBSCOhost Research Databases  Search Screen - Advanced Search  Database - CINAHL | 24 |
| S76 | S65 OR S66 OR S67 OR S68 OR S69 OR S70 OR S71 OR S72 OR S73 OR S74 OR S75 | Expanders - Apply related words; Apply equivalent subjects  Search modes - Boolean/Phrase | Interface - EBSCOhost Research Databases  Search Screen - Advanced Search  Database - CINAHL | 185,419 |
| S75 | TI overview# N2 guideline# OR AB overview# N2 guideline# | Expanders - Apply related words; Apply equivalent subjects  Search modes - Boolean/Phrase | Interface - EBSCOhost Research Databases  Search Screen - Advanced Search  Database - CINAHL | 171 |
| S74 | TI ( (critical or clinical or practice) N2 (path or paths or pathway or pathways or protocol*) ) OR AB ( (critical or clinical or practice) N2 (path or paths or pathway or pathways or protocol*) ) | Expanders - Apply related words; Apply equivalent subjects  Search modes - Boolean/Phrase | Interface - EBSCOhost Research Databases  Search Screen - Advanced Search  Database - CINAHL | 9,397 |
| S73 | TI recommendat* | Expanders - Apply related words; Apply equivalent subjects  Search modes - Boolean/Phrase | Interface - EBSCOhost Research Databases  Search Screen - Advanced Search  Database - CINAHL | 19,307 |
| S72 | TI consensus | Expanders - Apply related words; Apply equivalent subjects  Search modes - Boolean/Phrase | Interface - EBSCOhost Research Databases  Search Screen - Advanced Search  Database - CINAHL | 9,113 |
| S71 | TI CPG or CPGs | Expanders - Apply related words; Apply equivalent subjects  Search modes - Boolean/Phrase | Interface - EBSCOhost Research Databases  Search Screen - Advanced Search  Database - CINAHL | 358 |
| S70 | TI ( (practice or treatment* or clinical) W0 guideline* ) OR AB ( (practice or treatment* or clinical) W0 guideline* ) | Expanders - Apply related words; Apply equivalent subjects  Search modes - Boolean/Phrase | Interface - EBSCOhost Research Databases  Search Screen - Advanced Search  Database - CINAHL | 25,762 |
| S69 | TI standards or guideline or guidelines | Expanders - Apply related words; Apply equivalent subjects  Search modes - Boolean/Phrase | Interface - EBSCOhost Research Databases  Search Screen - Advanced Search  Database - CINAHL | 57,644 |
| S68 | TI ( (position W0 statement*) or (policy W0 statement*) or (practice W0 parameter*) or (best W0 practice*) ) OR AB ( (position W0 statement*) or (policy W0 statement*) or (practice W0 parameter*) or (best W0 practice*) ) | Expanders - Apply related words; Apply equivalent subjects  Search modes - Boolean/Phrase | Interface - EBSCOhost Research Databases  Search Screen - Advanced Search  Database - CINAHL | 26,847 |
| S67 | (MH "Practice Guidelines") | Expanders - Apply related words; Apply equivalent subjects  Search modes - Boolean/Phrase | Interface - EBSCOhost Research Databases  Search Screen - Advanced Search  Database - CINAHL | 81,241 |
| S66 | (MH "Consensus") | Expanders - Apply related words; Apply equivalent subjects  Search modes - Boolean/Phrase | Interface - EBSCOhost Research Databases  Search Screen - Advanced Search  Database - CINAHL | 5,025 |
| S65 | (MH "Critical Path") | Expanders - Apply related words; Apply equivalent subjects  Search modes - Boolean/Phrase | Interface - EBSCOhost Research Databases  Search Screen - Advanced Search  Database - CINAHL | 5,541 |
| S64 | S40 AND S63 | Expanders - Apply related words; Apply equivalent subjects  Search modes - Boolean/Phrase | Interface - EBSCOhost Research Databases  Search Screen - Advanced Search  Database - CINAHL | 26 |
| S63 | S47 OR S48 OR S49 OR S50 OR S51 OR S52 OR S53 OR S54 OR S55 OR S56 OR S57 OR S58 OR S59 OR S60 OR S61 OR S62 | Expanders - Apply related words; Apply equivalent subjects  Search modes - Boolean/Phrase | Interface - EBSCOhost Research Databases  Search Screen - Advanced Search  Database - CINAHL | 425,637 |
| S62 | TI trial | Expanders - Apply related words; Apply equivalent subjects  Search modes - Boolean/Phrase | Interface - EBSCOhost Research Databases  Search Screen - Advanced Search  Database - CINAHL | 117,223 |
| S61 | TI control* N2 group# OR AB control* N2 group# | Expanders - Apply related words; Apply equivalent subjects  Search modes - Boolean/Phrase | Interface - EBSCOhost Research Databases  Search Screen - Advanced Search  Database - CINAHL | 124,815 |
| S60 | (MH "Control Group") | Expanders - Apply related words; Apply equivalent subjects  Search modes - Boolean/Phrase | Interface - EBSCOhost Research Databases  Search Screen - Advanced Search  Database - CINAHL | 12,519 |
| S59 | TI control* N2 study OR AB control* N2 study | Expanders - Apply related words; Apply equivalent subjects  Search modes - Boolean/Phrase | Interface - EBSCOhost Research Databases  Search Screen - Advanced Search  Database - CINAHL | 78,448 |
| S58 | (MH "Historically Controlled Study") | Expanders - Apply related words; Apply equivalent subjects  Search modes - Boolean/Phrase | Interface - EBSCOhost Research Databases  Search Screen - Advanced Search  Database - CINAHL | 128 |
| S57 | TI pretest N5 posttest OR AB pretest N5 posttest | Expanders - Apply related words; Apply equivalent subjects  Search modes - Boolean/Phrase | Interface - EBSCOhost Research Databases  Search Screen - Advanced Search  Database - CINAHL | 5,360 |
| S56 | TI "pre-" N5 "post-" OR AB "pre-" N5 "post-" | Expanders - Apply related words; Apply equivalent subjects  Search modes - Boolean/Phrase | Interface - EBSCOhost Research Databases  Search Screen - Advanced Search  Database - CINAHL | 37,850 |
| S55 | (MH "Pretest-Posttest Design") | Expanders - Apply related words; Apply equivalent subjects  Search modes - Boolean/Phrase | Interface - EBSCOhost Research Databases  Search Screen - Advanced Search  Database - CINAHL | 45,983 |
| S54 | TI "time series" OR AB "time series" | Expanders - Apply related words; Apply equivalent subjects  Search modes - Boolean/Phrase | Interface - EBSCOhost Research Databases  Search Screen - Advanced Search  Database - CINAHL | 6,318 |
| S53 | (MH "Nonrandomized Trials") | Expanders - Apply related words; Apply equivalent subjects  Search modes - Boolean/Phrase | Interface - EBSCOhost Research Databases  Search Screen - Advanced Search  Database - CINAHL | 627 |
| S52 | (MH "Interrupted Time Series Analysis") | Expanders - Apply related words; Apply equivalent subjects  Search modes - Boolean/Phrase | Interface - EBSCOhost Research Databases  Search Screen - Advanced Search  Database - CINAHL | 575 |
| S51 | TI ( control* N3 ("before and after" or "before after") ) OR AB ( control* N3 ("before and after" or "before after") ) | Expanders - Apply related words; Apply equivalent subjects  Search modes - Boolean/Phrase | Interface - EBSCOhost Research Databases  Search Screen - Advanced Search  Database - CINAHL | 1,842 |
| S50 | (MH "Controlled Before-After Studies") | Expanders - Apply related words; Apply equivalent subjects  Search modes - Boolean/Phrase | Interface - EBSCOhost Research Databases  Search Screen - Advanced Search  Database - CINAHL | 198 |
| S49 | TI ( nRCT or "non-RCT" ) OR AB ( nRCT or "non-RCT" ) | Expanders - Apply related words; Apply equivalent subjects  Search modes - Boolean/Phrase | Interface - EBSCOhost Research Databases  Search Screen - Advanced Search  Database - CINAHL | 174 |
| S48 | TI ( nonrandom* or (non W0 random*) or (quasi W0 random*) or (quasi W0 experiment*) ) OR AB ( nonrandom* or (non W0 random*) or (quasi W0 random*) or (quasi W0 experiment*) ) | Expanders - Apply related words; Apply equivalent subjects  Search modes - Boolean/Phrase | Interface - EBSCOhost Research Databases  Search Screen - Advanced Search  Database - CINAHL | 23,669 |
| S47 | TI control* N2 trial OR AB control* N2 trial | Expanders - Apply related words; Apply equivalent subjects  Search modes - Boolean/Phrase | Interface - EBSCOhost Research Databases  Search Screen - Advanced Search  Database - CINAHL | 135,712 |
| S46 | S40 AND S45 | Expanders - Apply related words; Apply equivalent subjects  Search modes - Boolean/Phrase | Interface - EBSCOhost Research Databases  Search Screen - Advanced Search  Database - CINAHL | 33 |
| S45 | S41 OR S42 OR S43 OR S44 | Expanders - Apply related words; Apply equivalent subjects  Search modes - Boolean/Phrase | Interface - EBSCOhost Research Databases  Search Screen - Advanced Search  Database - CINAHL | 506,749 |
| S44 | TI trial | Expanders - Apply related words; Apply equivalent subjects  Search modes - Boolean/Phrase | Interface - EBSCOhost Research Databases  Search Screen - Advanced Search  Database - CINAHL | 117,223 |
| S43 | TI ( (singl* or doubl* or trebl* or tripl*) W0 (mask* or blind* or dumm*) ) OR AB ( (singl* or doubl* or trebl* or tripl*) W0 (mask* or blind* or dumm*) ) | Expanders - Apply related words; Apply equivalent subjects  Search modes - Boolean/Phrase | Interface - EBSCOhost Research Databases  Search Screen - Advanced Search  Database - CINAHL | 51,240 |
| S42 | TI ( randomi?ed or randomi?ation# or randomly or RCT or placebo* ) OR AB ( randomi?ed or randomi?ation# or randomly or RCT or placebo* ) | Expanders - Apply related words; Apply equivalent subjects  Search modes - Boolean/Phrase | Interface - EBSCOhost Research Databases  Search Screen - Advanced Search  Database - CINAHL | 344,683 |
| S41 | (MH "Randomized Controlled Trials") or (MH "Clinical Trials+") | Expanders - Apply related words; Apply equivalent subjects  Search modes - Boolean/Phrase | Interface - EBSCOhost Research Databases  Search Screen - Advanced Search  Database - CINAHL | 315,434 |
| S40 | S38 AND S39 | Expanders - Apply related words; Apply equivalent subjects  Search modes - Boolean/Phrase | Interface - EBSCOhost Research Databases  Search Screen - Advanced Search  Database - CINAHL | 632 |
| S39 |  | Limiters - Published Date: 20110101-20211231  Expanders - Apply related words; Apply equivalent subjects  Search modes - Boolean/Phrase | Interface - EBSCOhost Research Databases  Search Screen - Advanced Search  Database - CINAHL | 4,569,422 |
| S38 | S36 NOT S37 | Expanders - Apply related words; Apply equivalent subjects  Search modes - Boolean/Phrase | Interface - EBSCOhost Research Databases  Search Screen - Advanced Search  Database - CINAHL | 865 |
| S37 | (MH "Adult+") NOT ( (MH "Child+") or (MH "Adolescence+") or (MH "Minors") ) | Expanders - Apply related words; Apply equivalent subjects  Search modes - Boolean/Phrase | Interface - EBSCOhost Research Databases  Search Screen - Advanced Search  Database - CINAHL | 1,486,104 |
| S36 | S1 OR S2 OR S3 OR S4 OR S5 OR S6 OR S7 OR S8 OR S9 OR S10 OR S11 OR S12 OR S13 OR S14 OR S15 OR S16 OR S17 OR S18 OR S19 OR S20 OR S21 OR S22 OR S23 OR S24 OR S25 OR S26 OR S27 OR S28 OR S29 OR S30 OR S31 OR S32 OR S33 OR S34 OR S35 | Expanders - Apply related words; Apply equivalent subjects  Search modes - Boolean/Phrase | Interface - EBSCOhost Research Databases  Search Screen - Advanced Search  Database - CINAHL | 1,004 |
| S35 | TI ( (MPS W0 1#) or MPS1# or (MPS W0 I#) or MPSI# or "MPS1-H" or "MPSIH/S" or "MPS1H/S" or "MPS1-HS" or "MPS1-S" or MPSIS or "MPS 2" or MPS2 or "MPS II" or MPSII or (MPS W0 3#) or MPS3# or (MPS W0 III#) or MPSIII# or (MPS W0 4#) or MPS4# or (MPS W0 IV#) or MPSIV# or "MPS 6" or MPS6 or "MPS VI" or MPSVI or "MPS 7" or MPS7 or "MPS VII" or MPSVII or "MPS 9" or MPS9 or "MPS IX" or MPSIX ) OR AB ( (MPS W0 1#) or MPS1# or (MPS W0 I#) or MPSI# or "MPS1-H" or "MPSIH/S" or "MPS1H/S" or "MPS1-HS" or "MPS1-S" or MPSIS or "MPS 2" or MPS2 or "MPS II" or MPSII or (MPS W0 3#) or MPS3# or (MPS W0 III#) or MPSIII# or (MPS W0 4#) or MPS4# or (MPS W0 IV#) or MPSIV# or "MPS 6" or MPS6 or "MPS VI" or MPSVI or "MPS 7" or MPS7 or "MPS VII" or MPSVII or "MPS 9" or MPS9 or "MPS IX" or MPSIX ) | Expanders - Apply related words; Apply equivalent subjects  Search modes - Boolean/Phrase | Interface - EBSCOhost Research Databases  Search Screen - Advanced Search  Database - CINAHL | 266 |
| S34 | TI ( (myxedema* or myxoedema* or myxooedema* or myxo-oedema*) N3 (papular or papulos*) ) OR AB ( (myxedema* or myxoedema* or myxooedema* or myxo-oedema*) N3 (papular or papulos*) ) | Expanders - Apply related words; Apply equivalent subjects  Search modes - Boolean/Phrase | Interface - EBSCOhost Research Databases  Search Screen - Advanced Search  Database - CINAHL | 8 |
| S33 | TI ( (mucinosis or mucinous*) N3 (papular* or papulos*) ) OR AB ( (mucinosis or mucinous*) N3 (papular* or papulos*) ) | Expanders - Apply related words; Apply equivalent subjects  Search modes - Boolean/Phrase | Interface - EBSCOhost Research Databases  Search Screen - Advanced Search  Database - CINAHL | 15 |
| S32 | TI ( lichen N1 (fibromucinoidos* or fibro-mucinoidos* or myxedematos* or myxoedematos* or myxooedematos* or myxo-oedematos*) ) OR AB ( lichen N1 (fibromucinoidos* or fibro-mucinoidos* or myxedematos* or myxoedematos* or myxooedematos* or myxo-oedematos*) ) | Expanders - Apply related words; Apply equivalent subjects  Search modes - Boolean/Phrase | Interface - EBSCOhost Research Databases  Search Screen - Advanced Search  Database - CINAHL | 17 |
| S31 | TI ( scleromyxedem* or (sclero W0 myxedem*) ) OR AB ( scleromyxedem* or (sclero W0 myxedem*) ) | Expanders - Apply related words; Apply equivalent subjects  Search modes - Boolean/Phrase | Interface - EBSCOhost Research Databases  Search Screen - Advanced Search  Database - CINAHL | 67 |
| S30 | TI ( ("beta-glucuronidase" or GUSB) N2 deficien* ) OR AB ( ("beta-glucuronidase" or GUSB) N2 deficien* ) | Expanders - Apply related words; Apply equivalent subjects  Search modes - Boolean/Phrase | Interface - EBSCOhost Research Databases  Search Screen - Advanced Search  Database - CINAHL | 1 |
| S29 | TI ( sly N2 (disease# or syndrome#) ) OR AB ( sly N2 (disease# or syndrome#) ) | Expanders - Apply related words; Apply equivalent subjects  Search modes - Boolean/Phrase | Interface - EBSCOhost Research Databases  Search Screen - Advanced Search  Database - CINAHL | 9 |
| S28 | TI "mckusick 25320" OR AB "mckusick 25320" | Expanders - Apply related words; Apply equivalent subjects  Search modes - Boolean/Phrase | Interface - EBSCOhost Research Databases  Search Screen - Advanced Search  Database - CINAHL | 0 |
| S27 | TI ( ("arylsulfatase b" or "arylsulphatase b" or n-acetylgalactosamine-4-sulfat* or n-acetylgalactosamine-4-sulphat* or ARSB) N2 deficien* ) OR AB ( ("arylsulfatase b" or "arylsulphatase b" or n-acetylgalactosamine-4-sulfat* or n-acetylgalactosamine-4-sulphat* or ARSB) N2 deficien* ) | Expanders - Apply related words; Apply equivalent subjects  Search modes - Boolean/Phrase | Interface - EBSCOhost Research Databases  Search Screen - Advanced Search  Database - CINAHL | 7 |
| S26 | TI ( "maroteaux lamy" N2 (disease# or syndrome#) ) OR AB ( "maroteaux lamy" N2 (disease# or syndrome#) ) | Expanders - Apply related words; Apply equivalent subjects  Search modes - Boolean/Phrase | Interface - EBSCOhost Research Databases  Search Screen - Advanced Search  Database - CINAHL | 28 |
| S25 | TI ( eccentro#steochondrodysplas* or eccentro-osteochondrodysplas* or eccentro-osteo-chondrodysplasia* or eccentroosteo-chondrodysplas* ) OR AB ( eccentro#steochondrodysplas* or eccentro-osteochondrodysplas* or eccentro-osteo-chondrodysplasia* or eccentroosteo-chondrodysplas* ) | Expanders - Apply related words; Apply equivalent subjects  Search modes - Boolean/Phrase | Interface - EBSCOhost Research Databases  Search Screen - Advanced Search  Database - CINAHL | 0 |
| S24 | TI "familial osseous" W0 dystroph* OR AB "familial osseous" W0 dystroph* | Expanders - Apply related words; Apply equivalent subjects  Search modes - Boolean/Phrase | Interface - EBSCOhost Research Databases  Search Screen - Advanced Search  Database - CINAHL | 0 |
| S23 | TI ( (osteochondrodystroph* or osteo-chondrodystroph*) N1 deformans ) OR AB ( (osteochondrodystroph* or osteo-chondrodystroph*) N1 deformans ) | Expanders - Apply related words; Apply equivalent subjects  Search modes - Boolean/Phrase | Interface - EBSCOhost Research Databases  Search Screen - Advanced Search  Database - CINAHL | 0 |
| S22 | TI ( (kerato W0 sulfatur*) or (kerato W0 sulphatur*) or keratosulfatur* or keratosulphatur* ) OR AB ( (kerato W0 sulfatur*) or (kerato W0 sulphatur*) or keratosulfatur* or keratosulphatur* ) | Expanders - Apply related words; Apply equivalent subjects  Search modes - Boolean/Phrase | Interface - EBSCOhost Research Databases  Search Screen - Advanced Search  Database - CINAHL | 0 |
| S21 | TI ( ("galactosamine-6-sulfatase" or "galactosamine-6-sulphatase" or "n-acetylgalactosamine-6-sulfate sulfatase" or "n-acetylgalactosamine-6-sulphate sulphatase" or GALNS) N2 deficien* ) OR AB ( ("galactosamine-6-sulfatase" or "galactosamine-6-sulphatase" or "n-acetylgalactosamine-6-sulfate sulfatase" or "n-acetylgalactosamine-6-sulphate sulphatase" or GALNS) N2 deficien* ) | Expanders - Apply related words; Apply equivalent subjects  Search modes - Boolean/Phrase | Interface - EBSCOhost Research Databases  Search Screen - Advanced Search  Database - CINAHL | 4 |
| S20 | TI ( morquio* N2 (disease# or syndrome#) ) OR AB ( morquio* N2 (disease# or syndrome#) ) | Expanders - Apply related words; Apply equivalent subjects  Search modes - Boolean/Phrase | Interface - EBSCOhost Research Databases  Search Screen - Advanced Search  Database - CINAHL | 88 |
| S19 | TI ( ("n-acetylglucosamine-6-sulfate sulfatase" or "n-acetylglucosamine-6-sulphate sulphatase" or "glucosamine n-acetyl-6-sulfatase" or "glucosamine n-acetyl-6-sulphatase" or GNS) N2 deficien* ) OR AB ("n-acetylglucosamine-6-sulfate sulfatase" or "n-acetylglucosamine-6-sulphate sulphatase" or "glucosamine n-acetyl-6-sulfatase" or "glucosamine n-acetyl-6-sulphatase" or GNS) N2 deficien* ) | Expanders - Apply related words; Apply equivalent subjects  Search modes - Boolean/Phrase | Interface - EBSCOhost Research Databases  Search Screen - Advanced Search  Database - CINAHL | 1 |
| S18 | TI ( ("acetyl-CoA alpha-glucosaminide n-acetyltransferase" or "heparan-alpha-glucosaminide n-acetyltransferase" or HGSNAT) N2 deficien* ) OR AB ( ("acetyl-CoA alpha-glucosaminide n-acetyltransferase" or "heparan-alpha-glucosaminide n-acetyltransferase" or HGSNAT) N2 deficien* ) | Expanders - Apply related words; Apply equivalent subjects  Search modes - Boolean/Phrase | Interface - EBSCOhost Research Databases  Search Screen - Advanced Search  Database - CINAHL | 0 |
| S17 | TI ( (polydystrophic* or poly-dystrophic*) N2 (dwarf* or oligophreni* or oligo-phreni*) ) OR AB ( (polydystrophic* or poly-dystrophic*) N2 (dwarf* or oligophreni* or oligo-phreni*) ) | Expanders - Apply related words; Apply equivalent subjects  Search modes - Boolean/Phrase | Interface - EBSCOhost Research Databases  Search Screen - Advanced Search  Database - CINAHL | 0 |
| S16 | TI ( ("n-acetyl-alpha-d-glucosaminidase" or n-acetylglucosamine-6-sulfat* or n-acetylglucosamine-6-sulphat* or NAGLU) N2 deficien* ) OR AB ( ("n-acetyl-alpha-d-glucosaminidase" or n-acetylglucosamine-6-sulfat* or n-acetylglucosamine-6-sulphat* or NAGLU) N2 deficien* ) | Expanders - Apply related words; Apply equivalent subjects  Search modes - Boolean/Phrase | Interface - EBSCOhost Research Databases  Search Screen - Advanced Search  Database - CINAHL | 0 |
| S15 | TI ( (sanfilippo* or (san W0 filippo*)) N2 deficien* ) OR AB ( (sanfilippo* or (san W0 filippo*)) N2 deficien* ) | Expanders - Apply related words; Apply equivalent subjects  Search modes - Boolean/Phrase | Interface - EBSCOhost Research Databases  Search Screen - Advanced Search  Database - CINAHL | 0 |
| S14 | TI "mckusick 30990" OR AB "mckusick 30990" | Expanders - Apply related words; Apply equivalent subjects  Search modes - Boolean/Phrase | Interface - EBSCOhost Research Databases  Search Screen - Advanced Search  Database - CINAHL | 0 |
| S13 | TI heparitinuri* OR AB heparitinuri* | Expanders - Apply related words; Apply equivalent subjects  Search modes - Boolean/Phrase | Interface - EBSCOhost Research Databases  Search Screen - Advanced Search  Database - CINAHL | 0 |
| S12 | TI ( ("heparan sulfate" or "heparan sulphate" or "heparan sulfamidase" or "heparan sulphamidase" or "heparitin sulfate" or "heparitin sulphate" or "heparan-alpha-glucosaminide n-acetyltransferase") N2 (deficien* or (storage W0 disease#)) ) OR AB ( ("heparan sulfate" or "heparan sulphate" or "heparan sulfamidase" or "heparan sulphamidase" or "heparitin sulfate" or "heparitin sulphate" or "heparan-alpha-glucosaminide n-acetyltransferase") N2 (deficien* or (storage W0 disease#)) ) | Expanders - Apply related words; Apply equivalent subjects  Search modes - Boolean/Phrase | Interface - EBSCOhost Research Databases  Search Screen - Advanced Search  Database - CINAHL | 2 |
| S11 | TI hunter* W0 glossit?s OR AB hunter* W0 glossit?s | Expanders - Apply related words; Apply equivalent subjects  Search modes - Boolean/Phrase | Interface - EBSCOhost Research Databases  Search Screen - Advanced Search  Database - CINAHL | 1 |
| S10 | TI ( Hunter* N2 (disease# or syndrome#) ) OR AB ( Hunter* N2 (disease# or syndrome#) ) | Expanders - Apply related words; Apply equivalent subjects  Search modes - Boolean/Phrase | Interface - EBSCOhost Research Databases  Search Screen - Advanced Search  Database - CINAHL | 128 |
| S9 | TI ( (I2S or iduronate* or "iduronate 2" or sulfoiduronate* or sulfo-iduronate*) N2 deficien* ) OR AB ( (I2S or iduronate* or "iduronate 2" or sulfoiduronate* or sulfo-iduronate*) N2 deficien* ) | Expanders - Apply related words; Apply equivalent subjects  Search modes - Boolean/Phrase | Interface - EBSCOhost Research Databases  Search Screen - Advanced Search  Database - CINAHL | 5 |
| S8 | TI "mckusick 25280" OR AB "mckusick 25280" | Expanders - Apply related words; Apply equivalent subjects  Search modes - Boolean/Phrase | Interface - EBSCOhost Research Databases  Search Screen - Advanced Search  Database - CINAHL | 0 |
| S7 | TI ( lipochondrodystroph* or lipo-chondrodystroph* ) OR AB ( lipochondrodystroph* or lipo-chondrodystroph* ) | Expanders - Apply related words; Apply equivalent subjects  Search modes - Boolean/Phrase | Interface - EBSCOhost Research Databases  Search Screen - Advanced Search  Database - CINAHL | 0 |
| S6 | TI ( chondroosteodysplas* or chondro-osteodysplas* or chondroosteodystroph* or chondro-osteodystroph* or chondroosteoplas* or chondro-osteoplas* or (dysostos?s W0 multiplex) ) OR AB ( chondroosteodysplas* or chondro-osteodysplas* or chondroosteodystroph* or chondro-osteodystroph* or chondroosteoplas* or chondro-osteoplas* or (dysostos?s W0 multiplex) ) | Expanders - Apply related words; Apply equivalent subjects  Search modes - Boolean/Phrase | Interface - EBSCOhost Research Databases  Search Screen - Advanced Search  Database - CINAHL | 30 |
| S5 | TI ( "helmholtz harrington " N2 (disease# or syndrome#) ) OR AB ( "helmholtz harrington " N2 (disease# or syndrome#) ) | Expanders - Apply related words; Apply equivalent subjects  Search modes - Boolean/Phrase | Interface - EBSCOhost Research Databases  Search Screen - Advanced Search  Database - CINAHL | 0 |
| S4 | TI ( (Hurler* or Hurler-Scheie* or Pfaundler-Hurler* or Scheie* or Schleie*) N2 (disease# or syndrome#) ) OR AB ( (Hurler* or Hurler-Scheie* or Pfaundler-Hurler* or Scheie* or Schleie*) N2 (disease# or syndrome#) ) | Expanders - Apply related words; Apply equivalent subjects  Search modes - Boolean/Phrase | Interface - EBSCOhost Research Databases  Search Screen - Advanced Search  Database - CINAHL | 101 |
| S3 | TI ( ("alpha-l-iduronidase" or iduronidase) N2 (disease# or syndrome# or deficien*) ) OR AB ( ("alpha-l-iduronidase" or iduronidase) N2 (disease# or syndrome# or deficien*) ) | Expanders - Apply related words; Apply equivalent subjects  Search modes - Boolean/Phrase | Interface - EBSCOhost Research Databases  Search Screen - Advanced Search  Database - CINAHL | 13 |
| S2 | TI ( mucopolysaccharidos?s or gargolylis* or gargoylis* ) OR AB ( mucopolysaccharidos?s or gargolylis* or gargoylis* ) | Expanders - Apply related words; Apply equivalent subjects  Search modes - Boolean/Phrase | Interface - EBSCOhost Research Databases  Search Screen - Advanced Search  Database - CINAHL | 553 |
| S1 | (MH "Mucopolysaccharidoses+") | Expanders - Apply related words; Apply equivalent subjects  Search modes - Boolean/Phrase | Interface - EBSCOhost Research Databases  Search Screen - Advanced Search  Database - CINAHL | 640 |

**1.3 Draft Screening Form**

Dual review: articles screened by two independent reviewers

| **Phase One: Titles/Abstract Screening** | |
| --- | --- |
| Include | Exclude |
| Population:   - Children (≤18 years) or mixed population (children and adult) diagnosed with MPS   Study Design:   - Non-animal intervention studies of MPS - Clinical practice guidelines for MPS and other guidelines and recommendation papers related to MPS outcomes | Population:   - Adult population (>18 years) exclusively - Population not focused on MPS   Study Design:   - Animal/in-vitro studies - Non-intervention studies (i.e., observational studies, cross-sectional studies, case reports, case series, etc.)   Additional exclusions:   - Non-English - Published abstract only (e.g., conference proceeding) |
| **Phase Two: Full Text Screening** | |
| Include | Additional Exclusions |
| - If no exclusion criteria are met, include the article | - Wrong study design - Not a guideline - Abstract or conference proceeding - Published before 2011 - Age range unclear - Age group not specified - Adults (>18) exclusively - Not about patients diagnosed with MPS - Outcomes not reported separately for MPS patients - Outcomes not reported separately for children - No outcomes reported - Duplicate/already included - Not English - Full text not available - Other (specify) |
