# Supplementary material for: Establishing a core outcome set for mucopolysaccharidoses (MPS) in children: study protocol for a rapid literature review, candidate outcomes survey, and Delphi surveys
Source: Trials. 2021 Nov 17;22:816. doi: 10.1186/s13063-021-05791-8 (PMC8600749; doi:10.1186/s13063-021-05791-8)
Supplement: Supplementary file 3 — Additional file 3. COS-STAP checklist [file 13063_2021_5791_MOESM3_ESM.docx]

**Additional file 3: COS-STAP Statement Checklist**

| **Section** | | **Page number** |
| --- | --- | --- |
| TITLE/ABSTRACT | | |
| Title | Identify in the title that the paper describes the protocol for the planned development of a COS | 1 |
| Abstract | Provide a structured abstract | 3 |
| INTRODUCTION | | |
| Background and objectives | Describe the background and explain the rationale for developing the COS, and identify the reasons why a COS is needed and the potential barriers to its implementation | 4-7 |
|  | Describe the specific objectives with reference to developing a COS | 7 |
| Scope | Describe the health condition(s) and population(s) that will be covered by the COS | 4 |
|  | Describe the intervention(s) that will be covered by the COS | 10 |
|  | Describe the context of use for which the COS is to be applied | 10 |
| METHODS | | |
| Stakeholders | Describe the stakeholder groups to be involved in the COS development process, the nature of and rationale for their involvement and also how the individuals will be identified; this should cover involvement both as members of the research team and as participants in the study | 8, 13-16, 18-19, Figure 2 |
| Information sources | Describe the information sources that will be used to identify the list of outcomes. Outline the methods or reference other protocols/papers | 9-10 |
|  | Describe how outcomes may be dropped/combined, with reasons | 14 |
| Consensus process | Describe the plans for how the consensus process will be undertaken | 17-19 |
| Consensus definition | Describe the consensus definition | 18 |
|  | Describe the procedure for determining how outcomes will be added/combined/dropped from consideration during the consensus process | 18 |
| ANALYSIS | | |
| Outcome scoring/feedback | Describe how outcomes will be scored and summarised, describe how participants will receive feedback during the consensus process | 17-18 |
| Missing data | Describe how missing data will be handled during the consensus process | 18 |
| ETHICS AND DISSEMINATION | | |
| Ethics approval/informed consent | Describe any plans for obtaining research ethics committee/institutional review board approval in relation to the consensus process and describe how informed consent will be obtained (if relevant) | 23 |
| Dissemination | Describe any plans to communicate the results to study participants and COS users, inclusive of methods and timing of dissemination | 20, Figure 1 |
| ADMINISTRATIVE INFORMATION | | |
| Funders | Describe sources of funding, role of funders | 22 |
| Conflicts of interest | Describe any potential conflicts of interest within the study team and how they will be managed | 23 |
