# Supplementary material for: Establishing a core outcome set for mucopolysaccharidoses (MPS) in children: study protocol for a rapid literature review, candidate outcomes survey, and Delphi surveys
Source: Trials. 2021 Nov 17;22:816. doi: 10.1186/s13063-021-05791-8 (PMC8600749; doi:10.1186/s13063-021-05791-8)
Supplement: Supplementary file 4 — Additional file 4. Candidate outcomes survey [file 13063_2021_5791_MOESM4_ESM.docx]

**Survey: Establishing Outcomes for Mucopolysaccharidoses (MPS) in Children**

*Note: this was a web-based survey with automated skip patterns, so the questions did not appear exactly as shown below.*

Your participation in this survey is voluntary. We ask that you please answer all of the questions. However, if you feel uncomfortable answering questions about your/your child's age and MPS subtype, you may leave it blank.

**Information about you/your child**

1. Are you currently living in Canada?
   1. Yes
   2. No
      1. Thank you for your interest. At this time, only people living in Canada are eligible to participate in this survey. If you would like more information about INFORM RARE, please visit [www.informrare.ca](http://www.informrare.ca).
2. Please select the option that best describes you:
   1. Child or youth with MPS (aged 14-25 years)
   2. Parent or other adult caregiver of a child or youth with MPS (child is aged 18 years or younger)

*Questions 3-5 apply to those who selected 2a)*

*Questions 6-8 apply to those who selected 2b)*

1. What is your age in years?
   1. 14-16
   2. 17-19
   3. 20-22
   4. 23-25
2. Which MPS disorder have you been diagnosed with?
   1. I (Hurler, Hurler-Scheie, Scheie)
      1. Hurler syndrome
      2. Hurler-Scheie syndrome
      3. Scheie syndrome
      4. I am not sure
   2. II (Hunter)
   3. III (Sanfilippo)
      1. Sanfilippo syndrome type A
      2. Sanfilippo syndrome type B
      3. Sanfilippo syndrome type C
      4. Sanfilippo syndrome type D
      5. I am not sure
   4. IV (Morquio)
      1. Morquio syndrome type A
      2. Morquio syndrome type B
      3. I am not sure
   5. VI (Maroteaux-Lamy)
   6. VII (Sly)
   7. IX (Natowicz, Hyaluronidase deficiency)
   8. Other
      1. Please specify
3. At what age were you diagnosed with an MPS disorder?
   1. Before birth (prenatally)
   2. Under 1 year of age
   3. 1-2 years old
   4. 3-4 years old
   5. 5-11 years old
   6. 12-17 years old
   7. 18 years or older
   8. I am not sure
4. What is your child’s age in years?

*If you have more than one child diagnosed with MPS, please provide the ages of each affected child (check all that apply).*

- 1. Under 2
  2. 2-5
  3. 6-9
  4. 10-12
  5. 13-15
  6. 16-18

1. Which MPS disorder has your child been diagnosed with?

*If you have more than one child diagnosed with MPS, please provide the MPS subtype for each affected child (check all that apply).*

- 1. I (Hurler, Hurler-Scheie, Scheie)
     1. Hurler syndrome
     2. Hurler-Scheie syndrome
     3. Scheie syndrome
     4. I am not sure
  2. II (Hunter)
  3. III (Sanfilippo)
     1. Sanfilippo syndrome type A
     2. Sanfilippo syndrome type B
     3. Sanfilippo syndrome type C
     4. Sanfilippo syndrome type D
     5. I am not sure
  4. IV (Morquio)
     1. Morquio syndrome type A
     2. Morquio syndrome type B
     3. I am not sure
  5. VI (Maroteaux-Lamy)
  6. VII (Sly)
  7. IX (Natowicz, Hyaluronidase deficiency)
  8. Other
     1. Please specify

1. At what age was your child diagnosed with an MPS disorder?

*If you have more than one child diagnosed with MPS, please provide the age at diagnosis for each affected child (check all that apply).*

- 1. Before birth (prenatally)
  2. Under 1 year of age
  3. 1-2 years old
  4. 3-4 years old
  5. 5-11 years old
  6. 12-17 years old
  7. 18 years or older
  8. I am not sure

**Outcomes**

*Preamble for children/youth (those who selected 2a))*

**If you started taking a new treatment for MPS and you wanted to know whether this treatment was working for you, what sorts of outcomes would you like to see?**

An outcome is something that is important to a patient and that they would like to see change in response to a treatment. Outcomes can be related to symptoms or to changing the course of the disease and can include things like how you feel, what you can do, and observable symptoms. This could mean an improvement of something or preventing a later occurrence of something. For example, if you had chronic headaches, you might be interested in outcomes like your overall mood, your ability to take part in sports and physical activity, and the number of headaches you experience during a week.

You can put any outcomes that you think are important. There are no wrong answers!

**Please use the boxes below to list up to 3 outcomes for MPS. If you like, you can also let us know why you chose that outcome and what it means to you. If you would like to list more than 3 outcomes, additional space will be provided.**

*Preamble for parents/caregivers (those who selected 2b))*

**If your child started taking a new treatment for MPS and you wanted to know whether this treatment was working for them, what sorts of outcomes would you like to see?**

An outcome is something that researchers measure to study the effects of a treatment on patients. Outcomes can be related to symptoms or to changing the course of the disease and can include things like how you feel, what you can do, and observable symptoms. This could mean an improvement in something or preventing a later occurrence of something. For example, if you had chronic headaches, you might be interested in measuring your overall mood, your ability to take part in sports and physical activity, and the number of headaches you experience during a week.

You can put any outcomes that you think are important to measure. There are no wrong answers!

**Please use the boxes below to list up to 3 outcomes for MPS. If you like, you can also let us know why you chose that outcome and what it means to you. If you would like to list more than 3 outcomes, additional space will be provided.**

1. Outcome #1
   1. Optional: reason for choosing outcome #1
2. Outcome #2
   1. Optional: reason for choosing outcome #2
3. Outcome #3
   1. Optional: reason for choosing outcome #3
4. Would you like to list any additional outcomes that are important to you?

*Please use this space to list additional outcomes that are important to you. Feel free to list as many outcomes as you’d like.*

1. Do you have any additional comments about this research for the study team? (optional)
